# Estimating *F*_ST_ and kinship for arbitrary population structures

**DOI:** 10.1371/journal.pgen.1009241

**Published:** 2021-01-19

**Authors:** Alejandro Ochoa, John D. Storey

**Affiliations:** 1 Duke Center for Statistical Genetics and Genomics, Duke University, Durham, North Carolina, United States of America; 2 Department of Biostatistics and Bioinformatics, Duke University, Durham, North Carolina, United States of America; 3 Lewis-Sigler Institute for Integrative Genomics, Princeton University, Princeton, New Jersey, United States of America; Stanford University, UNITED STATES

## Abstract

*F*_ST_ and kinship are key parameters often estimated in modern population genetics studies in order to quantitatively characterize structure and relatedness. Kinship matrices have also become a fundamental quantity used in genome-wide association studies and heritability estimation. The most frequently-used estimators of *F*_ST_ and kinship are method-of-moments estimators whose accuracies depend strongly on the existence of simple underlying forms of structure, such as the independent subpopulations model of non-overlapping, independently evolving subpopulations. However, modern data sets have revealed that these simple models of structure likely do not hold in many populations, including humans. In this work, we analyze the behavior of these estimators in the presence of arbitrarily-complex population structures, which results in an improved estimation framework specifically designed for arbitrary population structures. After generalizing the definition of *F*_ST_ to arbitrary population structures and establishing a framework for assessing bias and consistency of genome-wide estimators, we calculate the accuracy of existing *F*_ST_ and kinship estimators under arbitrary population structures, characterizing biases and estimation challenges unobserved under their originally-assumed models of structure. We then present our new approach, which consistently estimates kinship and *F*_ST_ when the minimum kinship value in the dataset is estimated consistently. We illustrate our results using simulated genotypes from an admixture model, constructing a one-dimensional geographic scenario that departs nontrivially from the independent subpopulations model. Our simulations reveal the potential for severe biases in estimates of existing approaches that are overcome by our new framework. This work may significantly improve future analyses that rely on accurate kinship and *F*_ST_ estimates.

## Author summary

Kinship coefficients and *F*_ST_, which measure relatedness and population structure, respectively, are important quantities needed to accurately perform various analyses on genetic data, including genome-wide association studies and heritability estimation. However, existing estimators require restrictive assumptions of independence that are not met by real human and other datasets. In this work we find that existing estimators can be severely biased under reasonable scenarios, first by theoretically determining their properties, and then using an admixture simulation to illustrate our findings. In particular, we find that existing *F*_ST_ estimators are downwardly biased, and that existing kinship matrix estimators have related biases that are on average downward and of similar magnitude but vary for every pair of individuals. These insights led us to a new estimation framework for kinship and *F*_ST_ that is practically unbiased for any population structure, as demonstrated by theory and simulations. Our new approaches—available as open-source R packages—are easy to use and are more widely applicable than existing approaches, and they are likely to improve downstream analyses that require accurate kinship and *F*_ST_ estimates.

## Introduction

In population genetics studies, one is often interested in characterizing structure, genetic differentiation, and relatedness among individuals. Two quantities often considered in this context are *F*_ST_ and kinship. *F*_ST_ is a parameter that measures structure in a subdivided population, satisfying *F*_ST_ = 0 for an unstructured population and *F*_ST_ = 1 if every locus has become fixed for some allele in each subpopulation. More generally, *F*_ST_ is the probability that alleles drawn randomly from a subpopulation are “identical by descent” (IBD) relative to an ancestral population [1, 2]. The kinship coefficient is a measure of relatedness between individuals defined in terms of IBD probabilities, and it is closely related to *F*_ST_, since the mean kinship of the parents in a subpopulation is the *F*_ST_ of the following generation [1].

This work focuses on the estimation of *F*_ST_ and kinship from biallelic single-nucleotide polymorphism (SNP) data. Existing estimators can be classified into parametric estimators (methods that require a likelihood function) and non-parametric estimators (such as the method-of-moments estimators we focus on, which only require low-order moment equations). There are many likelihood approaches that estimate *F*_ST_ and kinship, but these are limited by assuming independent subpopulations or Normal approximations for *F*_ST_ [3–11] or totally outbred individuals for kinship [12, 13]. Additionally, more complete likelihood models such as that of Jacquard [14] are underdetermined for biallelic loci [15]. Non-parametric approaches such as those based on the method of moments are considerably more flexible and computationally tractable [16], so they are the natural choice to study arbitrary population structures.

The most frequently-used *F*_ST_ estimators are derived and justified under the “independent subpopulations model,” in which non-overlapping subpopulations evolved independently by splitting all at the same time from a common ancestral population. The Weir-Cockerham (WC) *F*_ST_ estimator assumes subpopulations of differing sample sizes and equal per-subpopulation *F*_ST_ relative to the common ancestral population [17]. The Weir-Hill *F*_ST_ estimator generalized WC for subpopulations with different *F*_ST_ values, and first considered arbitrary coancestry between subpopulations, resulting in estimates of a linearly-transformed *F*_ST_, namely $\left(F_{\text{ST}}-\tilde{\theta }\right)/\left(1-\tilde{\theta }\right)$ (where $\tilde{\theta }$ is the unknown mean coancestry value between subpopulations) [4, 18, 19]. Weir-Hill has further evolved into the Weir-Goudet approach, incorporating relatedness for subpopulations and individuals based on allele matching, also estimating a linearly-transformed *F*_ST_ [20–22]. Note that the Weir-Hill and Weir-Goudet approaches intended to estimate such linearly-transformed quantities, which may be negative, and they did not aim to estimate IBD probabilities [4, 18–22]; in contrast, our goal is to estimate IBD probabilities, which must be non-negative and valid probabilities. The “Hudson” *F*_ST_ estimator [23] assumes two subpopulations with different *F*_ST_ values. All of the previous *F*_ST_ estimators are ratio estimators derived using the method of moments to have unbiased numerators and denominators, which gives approximately unbiased ratio estimates when their assumptions are met [4, 17, 23]. We also evaluate BayeScan [10], which estimates population-specific *F*_ST_ values using a Bayesian model and the Dirichlet-Multinomial likelihood function—thus representing non-method-of-moments approaches—but which like other existing *F*_ST_ estimators also assumes that subpopulations are non-overlapping and evolve independently. These *F*_ST_ estimators are important contributions, used widely in the field.

Kinship coefficients are now commonly calculated in population genetics studies to capture structure and relatedness. Kinship is utilized in principal components analyses and linear-mixed effects models to correct for structure in Genome-Wide Association Studies (GWAS) [16, 24–30] and to estimate genome-wide heritability [31, 32]. Often absent in previous models is a clear identification and role of the ancestral population *T* that sets the scale of the kinship estimates used. Omission of *T* makes sense when kinship is estimated on an unstructured population (where only a few individual pairs are closely related; there *T* is the current population). Our more complete notation brings *T* to the fore and highlights its key role in kinship estimation and its applications. The most commonly-used kinship estimator [16, 27, 30–36] is also a method-of-moments estimator whose operating characteristics are largely unknown in the presence of structure. We show here that this popular estimator is accurate only when the average kinship is zero, which implies that the population must be unstructured.

The goal of our work is to consistently estimate IBD probabilities, namely kinship coefficients and *F*_ST_, for which there are currently no consistent estimators under general relatedness. Estimation of these as probabilities, as opposed to linearly-transformed quantities that may be negative, is important since the probabilistic definition of these parameters was required to derive their fundamental connections to many applications in genetics, including allele fixation [1, 2, 37], DNA forensics [3], and heritability [38, 39]. Although IBD probabilities are not absolute, but rather depend on the choice of ancestral population [40], their values become fixed upon agreeing to estimate them in terms of the Most Recent Common Ancestor (MRCA) population, which has long been the choice for models of *F*_ST_ [17, 23, 41] and kinship estimation from pedigrees [42, 43] or markers [12, 13].

Recent genome-wide studies have revealed that humans and other natural populations are structured in a complex manner that break the assumptions of the above estimators. Such complex population structures has been observed in several large human studies, such as the Human Genome Diversity Project [44, 45], the 1000 Genomes Project [46], Human Origins [47–49], and other contemporary [50–54] and archaic populations [55, 56]. We have also demonstrated that the global human population has a complex kinship matrix and no independent subpopulations [57–59]. Therefore, there is a need for innovative approaches designed for complex population structures. To this end, we reveal the operating characteristics of these frequently-used *F*_ST_ and kinship estimators in the presence of arbitrary forms of structure, which leads to a new estimation strategy for *F*_ST_ and kinship.

**Fig 1:**
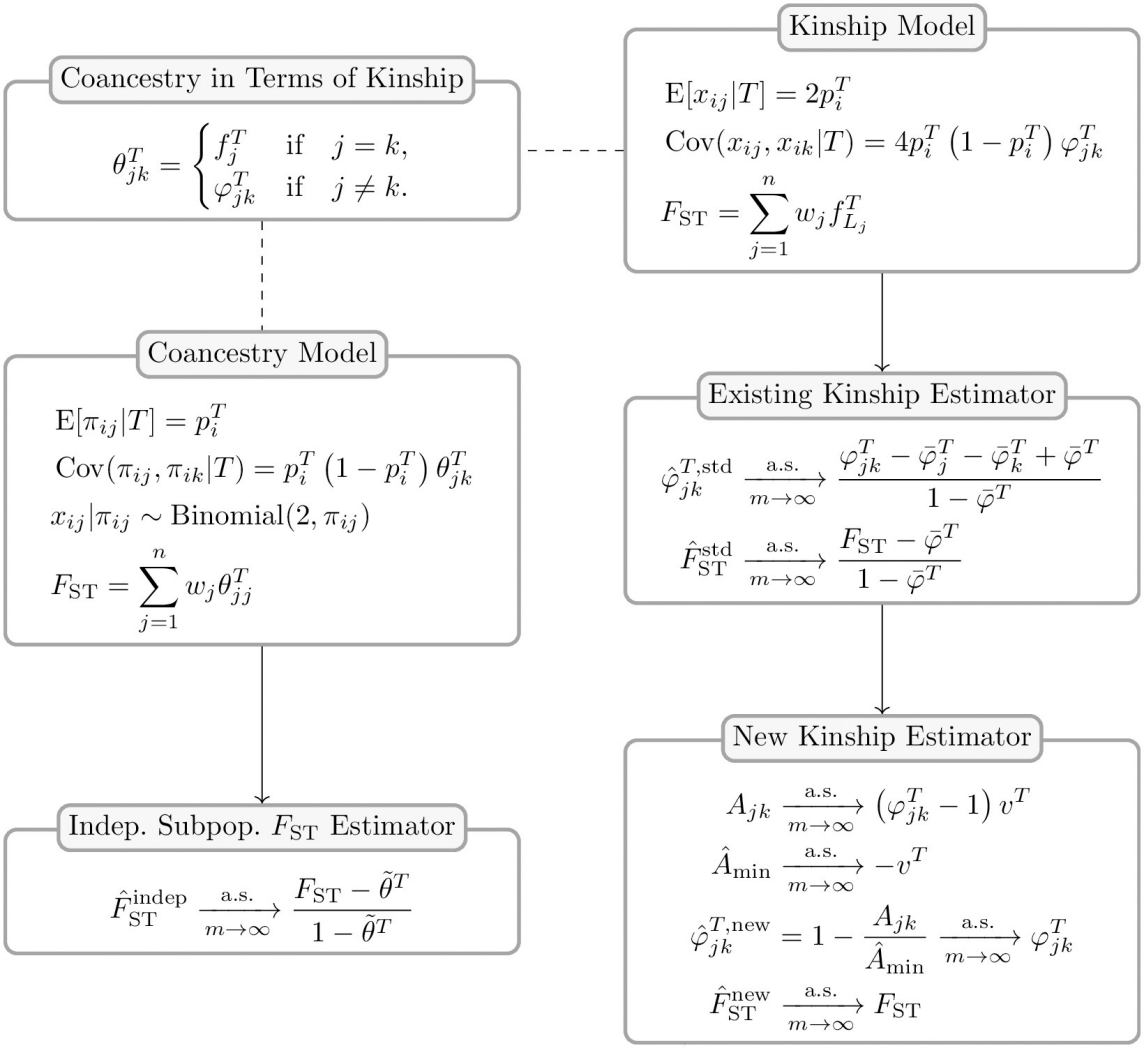
Accuracy of *F*_ST_ and kinship estimators: Overview of models and results. Our analysis is based on the generalized *F*_ST_ definition (section **The generalized *F*_ST_ for arbitrary population structures**) and two parallel models: the “Coancestry Model” for individual-specific allele frequencies (*π*_*ij*_), and the “Kinship Model” for genotypes (*x*_*ij*_). The “Coancestry in Terms of Kinship” panel connects kinship ($\varphi_{jk}^{T}$, $f_{j}^{T}$) and coancestry ($\theta_{jk}^{T}$) parameters (section **The kinship and coancestry models**). We use these models to study the accuracy of *F*_ST_ and kinship method-of-moment estimators under arbitrary population structures. The “Indep. Subpop. *F*_ST_ Estimator” panel shows the bias resulting from the misapplication of *F*_ST_ estimators for independent subpopulations (${\hat{F}}_{\text{ST}}^{\text{indep}}$) to arbitrary structures (section ***F*_ST_ estimation based on the independent subpopulations model**), as calculated under the coancestry model. The “Existing Kinship Estimator” panel shows the bias in the standard kinship model estimator (${\hat{\varphi }}_{jk}^{T,\text{std}}$) and its resulting plug-in *F*_ST_ estimator (${\hat{F}}_{\text{ST}}^{\text{std}}$; section **Characterizing a kinship estimator and its relationship to *F*_ST_**), as calculated under the kinship model. The “New Kinship Estimator” panel presents a new statistic *A*_*jk*_ that estimates kinship with a uniform bias, which together with a consistent estimator of its minimum value (${\hat{A}}_{\text{min}}$) results in our new kinship (${\hat{\varphi }}_{jk}^{T,\text{new}}$) and *F*_ST_ (${\hat{F}}_{\text{ST}}^{\text{new}}$) estimators, which are consistent under arbitrary population structure (section **A new approach for kinship and *F*_ST_ estimation**).

Here, we study existing *F*_ST_ and kinship method-of-moments estimators in models that allow for arbitrary population structures (see Fig 1 for an overview of the results). First, in section **The generalized *F*_ST_ for arbitrary population structures** we present the generalized definition of *F*_ST_ for arbitrary population structures [57]. In section **The kinship and coancestry models** we review the kinship model for genotype covariance [1, 14] and the coancestry model for individual-specific allele frequencies [57, 60, 61]. In section **Assessing the accuracy of genome-wide ratio estimators** we obtain new strong convergence results for a family of ratio estimators that includes the most common *F*_ST_ and kinship estimators. Next, we calculate the convergence values of these *F*_ST_ (section ***F*_ST_ estimation based on the independent subpopulations model**) and kinship (section **Characterizing a kinship estimator and its relationship to *F*_ST_**) estimators under arbitrary population structures, where we find biases that are not present under their original assumptions about structure (panels “Indep. Subpop. *F*_ST_ Estimator” and “Existing Kinship Estimator” in Fig 1). We characterize the limit of the standard kinship estimator, identifying complex biases or distortions, in agreement with recent work [21, 62].

In section **A new approach for kinship and *F*_ST_ estimation** we introduce a new approach for kinship and *F*_ST_ estimation for arbitrary population structures, and demonstrate the improved performance using a simple implementation of these estimators (panel “New Kinship Estimator” in Fig 1). There are two key innovations. First, based on the method of moments, we derive a statistic that estimates kinship coefficients up to a shared unknown scaling factor. Second, we propose a new condition, the identification of unrelated individual pairs in the data, which yields the value of the unknown scaling factor and enables the consistent estimation of kinship matrices and *F*_ST_. We present a simple implementation of this second estimator, based on taking the minimum average statistic value between subpopulations, which in section **Simulations evaluating *F*_ST_ and kinship estimators** is shown to perform well under some misspecification, namely in an admixture scenario that does not actually have subpopulations [63–65]. Elsewhere, we analyze the Human Origins and 1000 Genomes Project datasets with our novel kinship and *F*_ST_ estimation approach, where we demonstrate its coherence with the African Origins model, and illustrate the shortcomings of previous approaches in these complex data [59]. In summary, we identify a new approach for unbiased estimation of *F*_ST_ and kinship, and we provide new estimators that are nearly unbiased.

## Results

### The generalized *F*_ST_ for arbitrary population structures

The existing *F*_ST_ definition requires individuals to belong to discrete, non-overlapping subpopulations, so it must be generalized in order to apply to arbitrary population structures (such as the admixture model with individual-specific ancestry proportions considered in our simulations). Our generalized *F*_ST_ can be understood as a two-step strategy: (1) we define *F*_ST_ on a per-individual basis, and (2) we define *F*_ST_ for a group of individuals as a weighted average of the per-individual *F*_ST_ values [57].

The inbreeding coefficient $f_{j}^{T}$ of an individual *j* relative to an ancestral population *T* is defined as the probability that the two alleles at a random locus are *identical by descent* (IBD) [37]. Note that the ancestral population *T* determines what is IBD: only relationships since *T* count toward IBD. This *total* inbreeding coefficient ($f_{j}^{T}$) is the individual analog of Wright’s total inbreeding coefficient *F*_IT_, the latter of which is the mean $f_{j}^{T}$ over a group of individuals [2]. Wright partitioned *total* inbreeding (*F*_IT_) into *local* (*F*_IS_) and *structural* (*F*_ST_) coefficients defined by a subpopulation *S* that contains all individuals in question and evolved from the ancestral population *T*, so that *F*_IS_ is the inbreeding of individuals relative to *S* (as opposed to *T*) and *F*_ST_ is inbreeding of the subpopulation *S* relative to *T*, and these coefficients satisfy (1 − *F*_IT_) = (1 − *F*_IS_)(1 − *F*_ST_) [2]. In our generalized definitions for one individual *j*, we restrict the subpopulation of interest (*S*) to be *L*_*j*_, called the local subpopulation of *j*, which is the most recent subpopulation from which *j* drew its alleles. In this case, $f_{j}^{L_{j}}$ is the *local* inbreeding coefficient of *j* (always relative to its local subpopulation *L*_*j*_), and $f_{L_{j}}^{T}$ is the *structural* inbreeding coefficient of *j* (equal to the inbreeding of the subpopulation *L*_*j*_ relative to *T*), and being a special case of Wright’s equation, they also satisfy [57]

$$\begin{array}{c} (1-f_{j}^{T})=(1-f_{j}^{L_{j}})(1-f_{L_{j}}^{T}). \end{array}$$ (1)

Now we discuss estimating the three quantities we just introduced. First, the total inbreeding coefficient ($f_{j}^{T}$) should be estimated from the variance of genotypes, using the practically unbiased approach we introduce in this work. Second, note that the local inbreeding coefficient ($f_{j}^{L_{j}}$) corresponds to (non-population) family relatedness, so it can be taken to be the inbreeding calculated from a pedigree if it is available [42]. Note that estimation of the various inbreeding coefficients from pedigrees was the only approach available to Wright when he studied cattle and defined inbreeding and *F*_ST_ [2, 37]. Alternatively, in the absence of pedigrees, local inbreeding can be estimated from inferred self-IBD blocks or unusually-large runs of homozygosity [66–68]. Lastly, since the structural inbreeding coefficient ($f_{L_{j}}^{T}$) is given by the previous two quantities (solving from Eq (1)) by

$$\begin{array}{c} f_{L_{j}}^{T}=\frac{f_{j}^{T}-f_{j}^{L_{j}}}{1-f_{j}^{L_{j}}}, \end{array}$$ (2)

then we propose estimating $f_{L_{j}}^{T}$ using this equation, from the above estimates of $f_{j}^{T}$ and $f_{j}^{L_{j}}$.

As a toy example, suppose we estimate a total inbreeding coefficient of $f_{j}^{T}=0.15$ for a given individual whose parents are first cousins, then the pedigree expectation for its local inbreeding is $f_{j}^{L_{j}}=\frac{1}{16}=0.0625$, and the structural inbreeding (i.e. the *F*_ST_ of this individual) using Eq (2) is $f_{L_{j}}^{T}\approx 0.093$. However, if in the same example ($f_{j}^{T}=0.15$) the individual instead had parents who were second cousins, then $f_{j}^{L_{j}}=\frac{1}{64}\approx 0.0156$, then the structural estimate becomes $f_{L_{j}}^{T}\approx 0.137$, which is much closer to the total inbreeding value. Thus, when total inbreeding estimates are much larger than local inbreeding estimates, correcting for the latter via Eq (2) may not change the numerical estimate of structural inbreeding by a meaningful amount. Conversely, as the local inbreeding coefficient is reduced exponentially with the degree of relatedness of the parents ($f_{j}^{L_{j}}=\frac{1}{4^{n+1}}$ for *n*-th cousins), and as local inbreeding is required to be recent (to exclude population-level inbreeding), then sufficiently-accurate estimates of structural inbreeding can be obtained by estimating non-zero local inbreeding only for individuals with the most related parent pairs (above a certain degree of relatedness).

We define the generalized *F*_ST_ across *n* individuals as the weighted average of the per-individual structural inbreeding coefficients (*i.e.*, individual *F*_ST_ values) [57],

$$\begin{array}{c} F_{\text{ST}}=\sum_{j=1}^{n}w_{j}f_{L_{j}}^{T}, \end{array}$$ (3)

where *w*_*j*_ is the weight of individual *j* and the weights are required to sum to one and be non-negative. The above is a straightforward generalization of Wright’s *F*_ST_: if every individual *j* has *L*_*j*_ = *S* as its local subpopulation, then Eq (3) becomes $F_{\text{ST}}=\sum_{j=1}^{n}w_{j}f_{S}^{T}=f_{S}^{T}$, where $f_{S}^{T}$ is the inbreeding coefficient of subpopulation *S* relative to *T*, so it has the same meaning as Wright’s *F*_ST_ (the exact weights here do not matter as long as $\sum_{j=1}^{n}w_{j}=1$, as required). Moreover, if each individual *j* belongs to one of *K* subpopulations *S*_*u*_ (*u* ∈ {1, …, *K*}) and if subpopulations are weighted equally ($\sum_{j\in S_{u}}w_{j}=\frac{1}{K}$ for every *S*_*u*_), then Eq (3) becomes $F_{\text{ST}}=\frac{1}{K}\sum_{u=1}^{K}f_{S_{u}}^{T}$, so it equals the (unweighted) average subpopulation-specific *F*_ST_ (*i.e.*, $f_{S_{u}}^{T}$), which is the *F*_ST_ definition for multiple subpopulations prevalent in modern work [4, 21, 23]. The last case illustrates the need for weights, which above downweights individuals that belong to subpopulations with greater numbers of observations. In general, weights allow adjustment for skewed or unbalanced samples. However, in complicated scenarios without subpopulations and no obvious sampling biases, for simplicity we recommend using uniform weights ($w_{j}=\frac{1}{n}$) for the target generalized *F*_ST_.

In terms of total and local inbreeding coefficients (using Eq (2)), the generalized *F*_ST_ equals

$$\begin{array}{c} F_{\text{ST}}=\sum_{j=1}^{n}w_{j}\frac{f_{j}^{T}-f_{j}^{L_{j}}}{1-f_{j}^{L_{j}}}, \end{array}$$

which immediately suggests the estimation strategy when estimates of the total and local inbreeding coefficients are available. For simplicity, in the remainder of this work we shall consider only locally-outbred individuals ($f_{j}^{L_{j}}=0$ for all *j*), for which the generalized *F*_ST_ simply equals the weighted mean total inbreeding coefficient:

$$\begin{array}{c} F_{\text{ST}}=\sum_{j=1}^{n}w_{j}f_{j}^{T}. \end{array}$$ (4)

This greatly simplifies our discussion of bias for all of the *F*_ST_ estimators we analyzed; determining the statistical properties of local inbreeding estimators is beyond the scope of this work. Moreover, the assumption of locally-outbred individuals is satisfied in all of the simulations presented in this work.

### The kinship and coancestry models

The generalized *F*_ST_ above is given solely in terms of inbreeding coefficients. In order to establish our results and framework, it is necessary to consider kinship coefficients as well. The kinship coefficient is the extension of the inbreeding coefficient for a pair of individuals: the kinship coefficient $\varphi_{jk}^{T}$ of two individuals *j* and *k* relative to an ancestral population *T* is the probability that two alleles, chosen at random from each individual at a random locus, are IBD [1]. Note that the self-kinship coefficient is related to the inbreeding coefficient by $\varphi_{jj}^{T}=\frac{1}{2}(1+f_{j}^{T})$ [16].

Kinship coefficients determine the covariance structure of genotypes, which is the key to estimating kinship and *F*_ST_ from genotype data. We shall concentrate on biallelic variants, which include single-nucleotide polymorphisms, and are the dominant data from genotyping microarrays and whole-genome sequencing studies. We shall also restrict our attention to diploid organisms in this present work. Genotypes are encoded into variables *x*_*ij*_ for each locus *i* and individual *j* that count the number of alleles (dosage) of a given reference type, so for diploid organisms *x*_*ij*_ = 2 is homozygous for the reference allele, *x*_*ij*_ = 0 is homozygous for the alternative allele, and *x*_*ij*_ = 1 is heterozygous. Based on the definition of the IBD probabilities, the kinship model determines the mean and covariance structure of the genotype random variables at neutral loci [1, 2, 14, 16, 37]:

$$\begin{array}{cc} \text{E}[x_{ij}|T] & =2p_{i}^{T},\\ \text{Cov}(x_{ij},x_{ik}|T) & =4p_{i}^{T}(1-p_{i}^{T})\varphi_{jk}^{T}, \end{array}$$ (5)

where $p_{i}^{T}$ is the allele frequency at locus *i* in the ancestral population *T* and $0<p_{i}^{T}<1$.

The coancestry model resembles the kinship model, but it is formulated in terms of allele frequencies, which simplifies our analysis of *F*_ST_ estimators for subpopulations as well as yielding kinship coefficients under the admixture model we simulate from in this work. Let *π*_*ij*_ be the *individual-specific allele frequency* (IAF) at locus *i* for individual *j*, which is a real number between zero and one [60, 61]. Our coancestry model assumes that [57]

$$\begin{array}{c} \begin{array}{cc} E[\pi_{ij}|T] & =p_{i}^{T},\\ \mathrm{Cov}(\pi_{ij},\pi_{ik}|T) & =p_{i}^{T}(1-p_{i}^{T})\theta_{jk}^{T}, \end{array} \end{array}$$ (6)

where $\theta_{jk}^{T}$ is the coancestry coefficient between individuals *j* and *k* relative to the ancestral population *T*. This model is inspired by coancestry models for subpopulations common in the *F*_ST_ literature [4, 5, 21, 23], and exactly equals those models when subpopulation sizes go to infinity, in which case *j* and *k* index subpopulations rather than individuals, and *π*_*ij*_ is interpreted as the true allele frequency at locus *i* for subpopulation *j*.

The coancestry model connects to the kinship model under the additional assumption that the alleles of an individual *j* are drawn independently from its IAF,

$$\begin{array}{c} x_{ij}|\pi_{ij}∼\text{Binomial}\left(2,\pi_{ij}\right). \end{array}$$ (7)

In this case, marginalizing the intermediate IAF random variables (*π*_*ij*_) and matching the resulting genotype moments results in the following equivalence [57]:

$$\begin{array}{c} \theta_{jk}^{T}=\{\begin{array}{cc} f_{j}^{T} & \text{if}\;j=k,\\ \varphi_{jk}^{T} & \text{if}\;j\neq k. \end{array} \end{array}$$ (8)

The coancestry coefficient equals the kinship coefficient between two different individuals, but the self-coancestry coefficient equals the inbreeding coefficient (rather than the self-kinship coefficient). However, since in the coancestry model alleles are drawn independently conditional on the IAF in Eq (7), then the only structure present is the population structure, so these coancestry models cannot generate family structures, unlike the more general kinship model that also encompasses pedigrees. Therefore, despite Eq (8), the kinship and coancestry are not equivalent models except under the more restrictive assumptions of the coancestry model. Thus, individuals drawn from this model are always locally-outbred, so $\theta_{jj}^{T}=f_{L_{j}}^{T}$ also equals the structural inbreeding coefficient, and the generalized *F*_ST_ under the coancestry model is therefore

$$\begin{array}{c} F_{\text{ST}}=\sum_{j=1}^{n}w_{j}\theta_{jj}^{T}, \end{array}$$ (9)

which also generalizes previous definitions of *F*_ST_ under coancestry for subpopulations [4, 5, 21, 23]. The kinship and coancestry models, and their connection, is included in the overview Fig 1.

### Assessing the accuracy of genome-wide ratio estimators

In this section we change gears to focus on theoretical convergence properties of two broad estimator families. The resulting theory will be applied repeatedly to various *F*_ST_ and kinship estimators of interest in later sections.

Many *F*_ST_ and kinship coefficient method-of-moments estimators are *ratio estimators*, a general class of estimators that tends to be biased and to have no closed-form expectation [69]. In the *F*_ST_ literature, the expectation of a ratio is frequently approximated with a ratio of expectations [4, 17, 23]. Specifically, ratio estimators are often called “unbiased” if the ratio of expectations is unbiased, even though the ratio estimator itself may be biased [69]. Here we characterize the behavior of two ratio estimator families calculated from genome-wide data, known as “ratio-of-means” and “mean-of-ratios” estimators [23], detailing conditions where the previous approximation is justified and providing additional criteria to assess the accuracy of such estimators.

#### Ratio estimators

The general problem of forming ratio estimators involves random variables *a*_*i*_ and *b*_*i*_ calculated from genotypes at each locus *i*, such that E[*a*_*i*_] = *Ac*_*i*_ and E[*b*_*i*_] = *Bc*_*i*_ and the goal is to estimate $\frac{A}{B}$. *A* and *B* are constants shared across loci (given by *F*_ST_ or $\varphi_{jk}^{T}$), while *c*_*i*_ depends on the ancestral allele frequency $p_{i}^{T}$ and varies per locus. The problem is that the single-locus estimator $\frac{a_{i}}{b_{i}}$ is biased, since $E[\frac{a_{i}}{b_{i}}]\neq \frac{E[a_{i}]}{E[b_{i}]}=\frac{A}{B}$, which applies to ratio estimators in general [69]. Below we study two estimator families that combine large numbers of loci to better estimate $\frac{A}{B}$.

#### Convergence

The solution we recommend is the “ratio-of-means” estimator $\frac{{\hat{A}}_{m}}{{\hat{B}}_{m}}$, where ${\hat{A}}_{m}=\frac{1}{m}\sum_{i=1}^{m}a_{i}$, and ${\hat{B}}_{m}=\frac{1}{m}\sum_{i=1}^{m}b_{i}$, which is common for *F*_ST_ estimators [4, 17, 19, 23, 70]. Note that $E\left[{\hat{A}}_{m}\right]=A{\bar{c}}_{m}$ and $E\left[{\hat{B}}_{m}\right]=B{\bar{c}}_{m}$, where ${\bar{c}}_{m}=\frac{1}{m}\sum_{i=1}^{m}c_{i}$. We will assume bounded terms (|*a*_*i*_|, |*b*_*i*_| ≤ *C* for some finite *C*), a convergent ${\bar{c}}_{m}\to c$, and *Bc* ≠ 0, which are satisfied by common estimators. Given independent loci, we prove almost sure convergence to the desired quantity (S1 Text),

$$\begin{array}{c} \frac{{\hat{A}}_{m}}{{\hat{B}}_{m}}=\frac{\frac{1}{m}\sum_{i=1}^{m}a_{i}}{\frac{1}{m}\sum_{i=1}^{m}b_{i}}\overset{\text{a.s.}}{\underset{m\to \infty }{\to }}\frac{A}{B}, \end{array}$$ (10)

a strong result that implies $E[\frac{{\hat{A}}_{m}}{{\hat{B}}_{m}}]\to \frac{A}{B}$, justifying previous work [4, 17, 23]. Moreover, the error between these expectations scales with $\frac{1}{m}$ (S1 Text), just as for standard ratio estimators [69]. Although real loci are not independent due to genetic linkage, their dependence is very localized, so this estimator will perform well if the effective number of independent loci is large.

In order to test if a given ratio-of-means estimator converges to its ratio of expectations as in Eq (10), the following three conditions can be tested. (i) The expected values of each term *a*_*i*_, *b*_*i*_ must be calculated and shown to be of the form E[*a*_*i*_] = *Ac*_*i*_ and E[*b*_*i*_] = *Bc*_*i*_ for some *A* and *B* shared by all loci *i* and some *c*_*i*_ that may vary per locus *i* but must be shared by both E[*a*_*i*_], E[*b*_*i*_]. In the estimators we study, *A* and *B* are functions of IBD probabilities such as $\varphi_{jk}^{T}$ and *F*_ST_, while *c*_*i*_ is a function of $p_{i}^{T}$ only. (ii) The mean *c*_*i*_ must converge to a non-zero value for infinite loci. (iii) Both |*a*_*i*_|, |*b*_*i*_| ≤ *C* must be bounded for all *i* by some finite *C* (the estimators we study usually have *C* = 1 or *C* = 4). If these conditions are satisfied, then Eq (10) holds for independent loci and the *A* and *B* found in the first step. See the next section for an example application of this procedure to an *F*_ST_ estimator.

Another approach is the “mean-of-ratios” estimator $\frac{1}{m}\sum_{i=1}^{m}\frac{a_{i}}{b_{i}}$, used often to estimate kinship coefficients [16, 27, 30–35] and *F*_ST_ [46]. If each $\frac{a_{i}}{b_{i}}$ is biased, their average across loci will also be biased, even as *m* → ∞. However, if $E\left[\frac{a_{i}}{b_{i}}\right]\to \frac{A}{B}$ for all loci *i* = 1, …, *m* as the number of individuals *n* → ∞, and $\mathrm{Var}(\frac{a_{i}}{b_{i}})$ is bounded, then

$$\begin{array}{c} \frac{1}{m}\sum_{i=1}^{m}\frac{a_{i}}{b_{i}}\overset{\text{a.s.}}{\underset{n,m\to \infty }{\to }}\frac{A}{B}. \end{array}$$ (11)

Therefore, mean-of-ratios estimators must satisfy more restrictive conditions than ratio-of-means estimators, as well as large *n* (in addition to the large *m* needed by both estimators), to estimate $\frac{A}{B}$ well. We do not provide a procedure to test whether a given mean-of-ratios estimator converges as shown above.

### *F*_ST_ estimation based on the independent subpopulations model

Now that we have detailed how ratio estimators may be evaluated for their accuracy, we turn to existing estimators and assess their accuracy under arbitrary population structures. We study the *F*_ST_ estimators Weir-Cockerham (WC) [17], Weir-Hill [4], “Hudson” [23], and Weir-Goudet (equals HudsonK below for biallelic loci; S1 Text) [21]. The panel “Indep. Subpop. *F*_ST_ Estimator” in Fig 1 provides an overview of our results, which we detail in this section.

#### The *F*_ST_ estimator for independent subpopulations and infinite subpopulation sample sizes

The WC, Weir-Hill, and Hudson method-of-moments estimators have small sample size corrections that remarkably make them consistent (as the number of independent loci *m* goes to infinity) for finite numbers of individuals. However, these small sample corrections also make the estimators unnecessarily cumbersome for our purposes (see Methods, section **Previous *F*_ST_ estimators for the independent subpopulations model** for complete formulas). In order to illustrate clearly how these estimators behave, both under the independent subpopulations model and for arbitrary structure, here we construct simplified versions that assume infinite sample sizes per subpopulation (Methods, section **Previous *F*_ST_ estimators for the independent subpopulations model**). This simplification corresponds to eliminating statistical sampling, leaving only genetic sampling to analyze [71]. Note that our simplified estimator nevertheless illustrates the general behavior of the WC, Weir-Hill, and Hudson estimators under arbitrary structure, and the results are equivalent to those we would obtain under finite sample sizes of individuals. While the Hudson *F*_ST_ estimator compares two subpopulations [23], based on that work we derive a generalized “HudsonK” estimator for more than two subpopulations in Methods, section **Generalized HudsonK *F*_ST_ estimator**. Note that HudsonK, first derived in [58], also equals the Weir-Goudet *F*_ST_ estimator for subpopulations [21] when loci are biallelic, which was derived independently using allele matching (S1 Text).

Under infinite subpopulation sample sizes, the allele frequencies at each locus and every subpopulation are known. Let *j* ∈ {1, …, *n*} index subpopulations rather than individuals and *π*_*ij*_ be the true allele frequency in subpopulation *j* at locus *i*. Note that *π*_*ij*_ are not estimated allele frequencies, but rather true subpopulation allele frequencies; this abstraction does not result in a practical estimation approach, but it greatly simplifies understanding of bias for subpopulations in a setting where there there is no statistical sampling. Although in this analysis of *F*_ST_ estimators the *π*_*ij*_ values are applied to subpopulations, for coherence with our previous work we shall call them “individual-specific allele frequencies” (IAF) [60, 61]. Whether for individuals or subpopulations, the key assumption is that IAFs satisfy the coancestry model of Eq (6). In this special case of infinite subpopulation sample sizes, all of WC, Weir-Hill, and HudsonK simplify to the following *F*_ST_ estimator for independent subpopulations (“indep”; derived in Methods, section **Previous *F*_ST_ estimators for the independent subpopulations model**):

$${\hat{p}}_{i}^{T}=\frac{1}{n}\sum_{j=1}^{n}\pi_{ij},$$ (11)

$${\hat{\sigma }}_{i}^{2}=\frac{1}{n-1}\sum_{j=1}^{n}{\left(\pi_{ij}-{\hat{p}}_{i}^{T}\right)}^{2},$$ (12)

$${\hat{F}}_{\text{ST}}^{\text{indep}}=\frac{\sum_{i=1}^{m}{\hat{\sigma }}_{i}^{2}}{\sum_{i=1}^{m}{\hat{p}}_{i}^{T}(1-{\hat{p}}_{i}^{T})+\frac{1}{n}{\hat{\sigma }}_{i}^{2}}.$$ (13)

The goal is to estimate $F_{\text{ST}}=\frac{1}{n}\sum_{j=1}^{n}\theta_{jj}^{T}$, which is the special case of Eq (9) that weighs every subpopulation *j* equally ($w_{j}=\frac{1}{n}\;\forall j$).

#### *F*_ST_ estimation under the independent subpopulations model

Under the independent subpopulations model $\theta_{jk}^{T}=0$ for *j* ≠ *k*, where *T* is the most recent common ancestor (MRCA) population of the set of subpopulations. Note that the estimator in Eq (13) can be derived directly from Eq (6) and these assumptions using the method of moments (ignoring the existence of previous *F*_ST_ estimators; S1 Text). The expectations of the two recurrent terms in Eq (13) are

$$\begin{array}{cc} E[\frac{1}{m}\sum_{i=1}^{m}{\hat{\sigma }}_{i}^{2}|T] & ={\bar{p(1-p)}}^{T}F_{\text{ST}},\\ E[\frac{1}{m}\sum_{i=1}^{m}{\hat{p}}_{i}^{T}(1-{\hat{p}}_{i}^{T})|T] & ={\bar{p(1-p)}}^{T}(1-\frac{F_{\text{ST}}}{n}),\;\text{where}\\ {\bar{p(1-p)}}^{T} & =\frac{1}{m}\sum_{i=1}^{m}p_{i}^{T}(1-p_{i}^{T}). \end{array}$$

Eliminating ${\bar{p(1-p)}}^{T}$ and solving for *F*_ST_ in this system of equations recovers the estimator in Eq (13).

Before applying the convergence result in Eq (10), we test that the three conditions listed in section **Assessing the accuracy of genome-wide ratio estimators** are met. Condition (i): The locus *i* terms are $a_{i}={\hat{\sigma }}_{i}^{2}$ and $b_{i}={\hat{p}}_{i}^{T}(1-{\hat{p}}_{i}^{T})+\frac{1}{n}{\hat{\sigma }}_{i}^{2}$, which satisfy E[*a*_*i*_] = *Ac*_*i*_ and E[*b*_*i*_] = *Bc*_*i*_ with *A* = *F*_ST_, *B* = 1, and $c_{i}=p_{i}^{T}(1-p_{i}^{T})$. Condition (ii): ${\bar{c}}_{m}\to c=E[p_{i}^{T}(1-p_{i}^{T})|T]\neq 0$ over the $p_{i}^{T}$ distribution across loci. Condition (iii): Since $0\leq \pi_{ij},{\hat{p}}_{i}^{T}\leq 1$, then $0\leq {\hat{\sigma }}_{i}^{2}\leq 1$ and $0\leq {\hat{p}}_{i}^{T}(1-{\hat{p}}_{i}^{T})\leq \frac{1}{4}$, and since *n* ≥ 2, *C* = 1 bounds both |*a*_*i*_| and |*b*_*i*_|. Therefore, for independent loci,

$$\begin{array}{c} {\hat{F}}_{\text{ST}}^{\text{indep}}\overset{\text{a.s.}}{\underset{m\to \infty }{\to }}F_{\text{ST}}. \end{array}$$

#### *F*_ST_ estimation under arbitrary coancestry

Now we consider applying the independent subpopulations *F*_ST_ estimator to dependent subpopulations. The key difference is that now $\theta_{jk}^{T}\neq 0$ for every (*j*, *k*) will be assumed in our coancestry model in Eq (6), and now *T* may be either the MRCA population of all subpopulations or a more ancestral population. In this general setting, (*j*, *k*) may index either subpopulations or individuals. The two terms of ${\hat{F}}_{\text{ST}}^{\text{indep}}$ now satisfy

$$\begin{array}{cc} E[\frac{1}{m}\sum_{i=1}^{m}{\hat{\sigma }}_{i}^{2}|T] & ={\bar{p(1-p)}}^{T}(F_{\text{ST}}-{\bar{\theta }}^{T})\frac{n}{n-1},\\ E[\frac{1}{m}\sum_{i=1}^{m}{\hat{p}}_{i}^{T}(1-{\hat{p}}_{i}^{T})|T] & ={\bar{p(1-p)}}^{T}(1-{\bar{\theta }}^{T}), \end{array}$$

where ${\bar{\theta }}^{T}=\frac{1}{n^{2}}\sum_{j=1}^{n}\sum_{k=1}^{n}\theta_{jk}^{T}$ is the mean coancestry with uniform weights. There are two equations but three unknowns: *F*_ST_, ${\bar{\theta }}^{T}$, and ${\bar{p(1-p)}}^{T}$. The independent subpopulations model satisfies ${\bar{\theta }}^{T}=\frac{1}{n}F_{\text{ST}}$, which allows for the consistent estimation of *F*_ST_. Therefore, the new unknown ${\bar{\theta }}^{T}$ precludes consistent *F*_ST_ estimation without additional assumptions. As shown later, our additional assumption is that we can identify unrelated individuals in the data, which determines all unknowns. We defer our complete solution to this problem until kinship and its estimation challenges have been presented.

The *F*_ST_ estimator for independent subpopulations converges more generally to

$${\hat{F}}_{\text{ST}}^{\text{indep}}\overset{\text{a.s.}}{\underset{m\to \infty }{\to }}\frac{F_{\text{ST}}-{\tilde{\theta }}^{T}}{1-{\tilde{\theta }}^{T}},$$ (14)

(the conclusion of panel “Indep. Subpop. *F*_ST_ Estimator” in Fig 1), where

$$\begin{array}{c} {\tilde{\theta }}^{T}=\frac{1}{n-1}(n{\bar{\theta }}^{T}-F_{\text{ST}})=\frac{1}{n(n-1)}\sum_{j\neq k}\theta_{jk}^{T} \end{array}$$

is the average of all between-subpopulation coancestry coefficients, in agreement with related calculations regarding the WC and Weir-Hill estimators [4, 21]. Therefore, under arbitrary structure the independent subpopulations estimator’s bias is due to the coancestry between subpopulations. While the limit in Eq (14) appears to vary depending on the choice of *T*, it is in fact a constant with respect to *T* (proof in S1 Text).

Since $\frac{1}{n}F_{\text{ST}}\leq {\bar{\theta }}^{T}\leq F_{\text{ST}}$ (S1 Text), this estimator has a downward bias in the general setting: it is asymptotically unbiased (${\hat{F}}_{\text{ST}}^{\text{indep}}\overset{\text{a.s.}}{\underset{m\to \infty }{\to }}F_{\text{ST}}$) only when ${\bar{\theta }}^{T}=\frac{1}{n}F_{\text{ST}}$, while bias is maximal when ${\bar{\theta }}^{T}=F_{\text{ST}}$, where ${\hat{F}}_{\text{ST}}^{\text{indep}}\overset{\text{a.s.}}{\underset{m\to \infty }{\to }}0$. For example, if $\min\;\theta_{jk}^{T}=0$ so the MRCA population *T* is fixed, but *n* is large and $\theta_{jk}^{T}\approx F_{\text{ST}}$ for most pairs of subpopulations, then ${\bar{\theta }}^{T}\approx F_{\text{ST}}$ as well, and ${\hat{F}}_{\text{ST}}^{\text{indep}}\approx 0$. Therefore, the magnitude of the bias of ${\hat{F}}_{\text{ST}}^{\text{indep}}$ is unknown if ${\bar{\theta }}^{T}$ is unknown, and small ${\hat{F}}_{\text{ST}}^{\text{indep}}$ estimates may arise even if *F*_ST_ is very large.

#### Coancestry estimation as a method of moments

Since the generalized *F*_ST_ is given by coancestry coefficients $\theta_{jj}^{T}$ in Eq (9), a new *F*_ST_ estimator could be derived from estimates of $\theta_{jj}^{T}$. Here we attempt to define a method-of-moments estimator for $\theta_{jk}^{T}$, and find an underdetermined estimation problem, just as for *F*_ST_. This is consistent with IBD parameters in general requiring a reference population to be determined [40], whereas in this subsection this reference population is unspecified.

Given IAFs and the coancestry model of Eq (6), the first and second moments that average across loci are

$$E[\frac{1}{m}\sum_{i=1}^{m}\pi_{ij}|T]={\bar{p}}^{T},$$ (15)

$$E[\frac{1}{m}\sum_{i=1}^{m}\pi_{ij}\pi_{ik}|T]={\bar{p^{2}}}^{T}+{\bar{p(1-p)}}^{T}\theta_{jk}^{T},$$ (16)

where ${\bar{p}}^{T}=\frac{1}{m}\sum_{i=1}^{m}p_{i}^{T}$, ${\bar{p^{2}}}^{T}=\frac{1}{m}\sum_{i=1}^{m}{\left(p_{i}^{T}\right)}^{2}$, and ${\bar{p(1-p)}}^{T}$ is as before.

Suppose first that only $\theta_{jj}^{T}$ are of interest. There are *n* estimators given by Eq (16) with *j* = *k*, each corresponding to an unknown $\theta_{jj}^{T}$. However, all these estimators share two nuisance parameters: ${\bar{p}}^{T}$ and ${\bar{p^{2}}}^{T}$. While ${\bar{p}}^{T}$ can be estimated from Eq (15), there are no more equations left to estimate ${\bar{p^{2}}}^{T}$, so this system is underdetermined. The estimation problem remains underdetermined if all $\frac{n(n+1)}{2}$ estimators in Eq (16) are considered rather than only the *j* = *k* cases. Therefore, we cannot estimate coancestry coefficients consistently using only the first two moments without additional assumptions.

### Characterizing a kinship estimator and its relationship to *F*_ST_

Given the biases we see for ${\hat{F}}_{\text{ST}}^{\text{indep}}$ under arbitrary structures in the previous section, we now turn to the generalized definition of *F*_ST_ and pursue an estimate of it. Recall that our generalized *F*_ST_ in Eq (3) is defined in terms of inbreeding coefficients, which are a special case of the kinship coefficient. Kinship coefficients also determine the bias of ${\hat{F}}_{\text{ST}}^{\text{indep}}$ in Eq (14) (since coancestry and kinship coefficients are closely related: see panel “Coancestry in Terms of Kinship” in Fig 1). Therefore, we will consider estimates of kinship and inbreeding in this section. Estimating kinship is also important for GWAS approaches that control for population structure [16, 24–35, 72, 73].

In this section, we focus on a standard kinship method-of-moments estimator and calculate its limit for the first time (panel “Existing Kinship Estimator” in Fig 1). We study estimators that use genotypes or IAFs, and construct *F*_ST_ estimators from their kinship estimates. We find biases comparable to those of ${\hat{F}}_{\text{ST}}^{\text{indep}}$ in the previous section, and define unbiased *F*_ST_ estimators that require knowing the mean kinship or coancestry, or its proportion relative to *F*_ST_. The results of this section directly motivate and help construct our new kinship and *F*_ST_ estimation approach in the following section.

#### Characterization of the standard kinship estimator

Here we analyze a standard kinship estimator that is frequently used [16, 27, 30–36]. We generalize this estimator to use weights in estimating the ancestral allele frequencies, and we write it as a ratio-of-means estimator due to the favorable theoretical properties of this format as detailed in the earlier section **Assessing the accuracy of genome-wide ratio estimators**:

$${\hat{p}}_{i}^{T}=\frac{1}{2}\sum_{j=1}^{n}w_{j}x_{ij},$$ (17)

$${\hat{\varphi }}_{jk}^{T,\text{std}}=\frac{\sum_{i=1}^{m}\left(x_{ij}-2{\hat{p}}_{i}^{T})(x_{ik}-2{\hat{p}}_{i}^{T}\right)}{4\sum_{i=1}^{m}{\hat{p}}_{i}^{T}(1-{\hat{p}}_{i}^{T})}.$$ (18)

The estimator in Eq (18) resembles the sample covariance estimator applied to genotypes, but centers by locus *i* rather than by individuals *j* and *k*, and normalizes using estimates of $4p_{i}^{T}(1-p_{i}^{T})$. We derive Eq (18) directly using the method of moments in S1 Text. The weights in Eq (17) must satisfy *w*_*j*_ > 0 and $\sum_{j=1}^{n}w_{j}=1$, so that $0\leq {\hat{p}}_{i}^{T}\leq 1$ and $E[{\hat{p}}_{i}^{T}|T]=p_{i}^{T}$.

Utilizing the kinship model for genotypes from Eq (5), we find that Eq (18) converges to

$$\begin{array}{c} {\hat{\varphi }}_{jk}^{T,\text{std}}\overset{\text{a.s.}}{\underset{m\to \infty }{\to }}\frac{\varphi_{jk}^{T}-{\bar{\varphi }}_{j}^{T}-{\bar{\varphi }}_{k}^{T}+{\bar{\varphi }}^{T}}{1-{\bar{\varphi }}^{T}}, \end{array}$$ (19)

where ${\bar{\varphi }}_{j}^{T}=\sum_{k^{\prime }=1}^{n}w_{k^{\prime }}\varphi_{jk^{\prime }}^{T}$ and ${\bar{\varphi }}^{T}=\sum_{j^{\prime }=1}^{n}\sum_{k^{\prime }=1}^{n}w_{j^{\prime }}w_{k^{\prime }}\varphi_{j^{\prime }k^{\prime }}^{T}$, which agrees with related derivations [21, 62]. (This is the conclusion of panel “Existing Kinship Estimator” in Fig 1; see S1 Text for intermediate calculations that lead to Eq (19).) Therefore, the bias of ${\hat{\varphi }}_{jk}^{T,\text{std}}$ varies per pair of individuals *j* and *k*. Analogous distortions have been observed for sample covariances of genotypes [74]. The limit of ${\hat{\varphi }}_{jk}^{T,\text{std}}$ in Eq (19) is constant with respect to *T* (proof in S1 Text). Similarly, inbreeding coefficient estimates derived from Eq (18) converge to

$$\begin{array}{c} {\hat{f}}_{j}^{T,\text{std}}=2{\hat{\varphi }}_{jj}^{T}-1\overset{\text{a.s.}}{\underset{m\to \infty }{\to }}\frac{f_{j}^{T}-4{\bar{\varphi }}_{j}^{T}+3{\bar{\varphi }}^{T}}{1-{\bar{\varphi }}^{T}}. \end{array}$$ (20)

The difference between the bias of ${\hat{\varphi }}_{jk}^{T,\text{std}}$ for *j* ≠ *k* in Eq (19) and ${\hat{f}}_{j}^{T,\text{std}}$ in Eq (20) is visible in the kinship estimates shown toward the end of the results section. The limits of the ratio-of-means versions of two more $f_{j}^{T}$ estimators [32] are, if ${\hat{p}}_{i}^{T}$ uses Eq (17),

$$\begin{array}{c} \begin{array}{cc} {\hat{f}}_{j}^{T,\text{stdII}} & =1-\frac{\sum_{i=1}^{m}x_{ij}(2-x_{ij})}{2\sum_{i=1}^{m}{\hat{p}}_{i}^{T}(1-{\hat{p}}_{i}^{T})}\overset{\text{a.s.}}{\underset{m\to \infty }{\to }}\frac{f_{j}^{T}-{\bar{\varphi }}^{T}}{1-{\bar{\varphi }}^{T}},\\ {\hat{f}}_{j}^{T,\text{stdIII}} & =\frac{\sum_{i=1}^{m}x_{ij}^{2}-(1+2{\hat{p}}_{i}^{T})x_{ij}+2{\left({\hat{p}}_{i}^{T}\right)}^{2}}{2\sum_{i=1}^{m}{\hat{p}}_{i}^{T}(1-{\hat{p}}_{i}^{T})}\overset{\text{a.s.}}{\underset{m\to \infty }{\to }}\frac{f_{j}^{T}+{\bar{\varphi }}^{T}-2{\bar{\varphi }}_{j}^{T}}{1-{\bar{\varphi }}^{T}}. \end{array} \end{array}$$ (21)

The estimators in Eqs (18) and (21) are unbiased when ${\hat{p}}_{i}^{T}$ is replaced by $p_{i}^{T}$ [16, 32, 36], and are consistent when ${\hat{p}}_{i}^{T}$ is consistent [60]. Surprisingly, ${\hat{p}}_{i}^{T}$ in Eq (17) is not consistent (it does not converge almost surely to $p_{i}^{T}$) for arbitrary population structures, which is at the root of the bias in Eqs (19) to (21). In particular, although ${\hat{p}}_{i}^{T}$ is unbiased, its variance (S1 Text, and some special cases shown elsewhere, *e.g.*, [19]),

$$\begin{array}{c} \mathrm{Var}({\hat{p}}_{i}^{T}|T)=p_{i}^{T}(1-p_{i}^{T}){\bar{\varphi }}^{T}, \end{array}$$ (22)

may be asymptotically non-zero as *n* → ∞, since $p_{i}^{T}\in \left(0,1\right)$ is fixed and $\lim_{n\to \infty }{\bar{\varphi }}^{T}$ may take on any value between zero and one for arbitrary population structures. Further, ${\bar{\varphi }}^{T}\to 0$ as *n* → ∞ if and only if $\varphi_{jk}^{T}=0$ for almost all pairs of individuals (*j*, *k*). These observations hold for any weights such that $w_{j}>0,\sum_{j=1}^{n}w_{j}=1$. An important consequence is that the plug-in estimate of $p_{i}^{T}(1-p_{i}^{T})$ is biased (S1 Text),

$$\begin{array}{c} E[{\hat{p}}_{i}^{T}(1-{\hat{p}}_{i}^{T})|T]=p_{i}^{T}(1-p_{i}^{T})(1-{\bar{\varphi }}^{T}), \end{array}$$

which is present in all estimators we have studied.

#### Estimation of coancestry coefficients from IAFs

Here we form a coancestry coefficient estimator analogous to Eq (18) but using IAFs. Assuming the moments in Eq (6), this estimator and its limit are

$${\hat{p}}_{i}^{T}=\sum_{j=1}^{n}w_{j}\pi_{ij},$$ (23)

$${\hat{\theta }}_{jk}^{T,\text{std}}=\frac{\sum_{i=1}^{m}(\pi_{ij}-{\hat{p}}_{i}^{T})(\pi_{ik}-{\hat{p}}_{i}^{T})}{\sum_{i=1}^{m}{\hat{p}}_{i}^{T}(1-{\hat{p}}_{i}^{T})}\overset{\text{a.s.}}{\underset{m\to \infty }{\to }}\frac{\theta_{jk}^{T}-{\bar{\theta }}_{j}^{T}-{\bar{\theta }}_{k}^{T}+{\bar{\theta }}^{T}}{1-{\bar{\theta }}^{T}},$$ (24)

where ${\bar{\theta }}_{j}^{T}=\sum_{k=1}^{n}w_{k}\theta_{jk}^{T}$ and ${\bar{\theta }}^{T}=\sum_{j=1}^{n}\sum_{k=1}^{n}w_{j}w_{k}\theta_{jk}^{T}$ are analogous to ${\bar{\varphi }}_{j}^{T}$ and ${\bar{\varphi }}^{T}$. Eq (23) generalizes Eq (11) for arbitrary weights. Thus, use of IAFs does not ameliorate the estimation problems we have identified for genotypes. Like Eq (22), ${\hat{p}}_{i}^{T}$ in Eq (23) is not consistent because $\text{Var}({\hat{p}}_{i}^{T}|T)=p_{i}^{T}(1-p_{i}^{T}){\bar{\theta }}^{T}$ may not converge to zero for arbitrary population structures, which causes the bias observed in Eq (24).

#### *F*_ST_ estimator based on the standard kinship estimator

Since the generalized *F*_ST_ is defined as a mean inbreeding coefficient in Eq (3), here we study the *F*_ST_ estimator constructed as ${\hat{F}}_{\text{ST}}^{\text{std}}=\sum_{j=1}^{n}w_{j}{\hat{f}}_{j}^{T,\text{std}}$ where ${\hat{f}}_{j}^{T,\text{std}}$ is the inbreeding estimator derived from the standard kinship estimator. Although ${\hat{f}}_{j}^{T,\text{std}}$ is biased, we nevertheless plug it into our definition of *F*_ST_ so that we may study how bias manifests. Note that we do not recommend utilizing this *F*_ST_ estimator in practice, but we find these results informative for identifying how to proceed in deriving new estimators in the following section.

Remarkably, the three $f_{j}^{T}$ estimators in Eqs (20) and (21) give exactly the same plug-in ${\hat{F}}_{\text{ST}}^{\text{std}}$ if the weights in *F*_ST_ and ${\hat{p}}_{i}^{T}$ in Eq (17) match, namely

$$\begin{array}{c} {\hat{F}}_{\text{ST}}^{\text{std}}=\sum_{j=1}^{n}w_{j}{\hat{f}}_{j}^{T,\text{std}}=\frac{\sum_{i=1}^{m}\sum_{j=1}^{n}w_{j}{\left(x_{ij}-2{\hat{p}}_{i}^{T}\right)}^{2}}{2\sum_{i=1}^{m}{\hat{p}}_{i}^{T}(1-{\hat{p}}_{i}^{T})}-1\overset{\text{a.s.}}{\underset{m\to \infty }{\to }}\frac{F_{\text{ST}}-{\bar{\varphi }}^{T}}{1-{\bar{\varphi }}^{T}}, \end{array}$$ (25)

where the limit assumes locally-outbred individuals so Eq (4) holds. The analogous *F*_ST_ estimator for IAFs and its limit are

$$\begin{array}{c} {\hat{F}}_{\text{ST}}^{\text{std}}=\sum_{j=1}^{n}w_{j}{\hat{\theta }}_{jj}^{T,\text{std}}=\frac{\sum_{i=1}^{m}\sum_{j=1}^{n}w_{j}{\left(\pi_{ij}-{\hat{p}}_{i}^{T}\right)}^{2}}{\sum_{i=1}^{m}{\hat{p}}_{i}^{T}(1-{\hat{p}}_{i}^{T})}\overset{\text{a.s.}}{\underset{m\to \infty }{\to }}\frac{F_{\text{ST}}-{\bar{\theta }}^{T}}{1-{\bar{\theta }}^{T}}. \end{array}$$ (26)

The estimators in Eqs (25) and (26) for individuals and their limits resemble those of classical *F*_ST_ estimators for populations of the form $\frac{\sigma_{p}^{2}}{\bar{p}\left(1-\bar{p}\right)}$ [4, 5]. ${\hat{F}}_{\text{ST}}^{\text{std}}$ in Eq (26) for subpopulations *j* with uniform weight and one locus is also *G*_ST_ for two alleles [75]. Compared to ${\hat{F}}_{\text{ST}}^{\text{indep}}$ in Eq (13), ${\hat{F}}_{\text{ST}}^{\text{std}}$ in Eq (26) admits arbitrary weights and, by forgoing bias correction under the independent subpopulations model, is a simpler target of study.

Like ${\hat{F}}_{\text{ST}}^{\text{indep}}$ in Eq (13), ${\hat{F}}_{\text{ST}}^{\text{std}}$ in Eqs (25) and (26) are downwardly biased since $0\leq {\bar{\varphi }}^{T},{\bar{\theta }}^{T}$. ${\hat{F}}_{\text{ST}}^{\text{std}}$ in Eq (26) may converge arbitrarily close to zero since ${\bar{\theta }}^{T}$ can be arbitrarily close to *F*_ST_ (S1 Text). Moreover, although ${\bar{\varphi }}^{T}\approx {\bar{\theta }}^{T}$ for large *n* (see Eq (8) and panel “Coancestry in Terms of Kinship” in Fig 1), in extreme cases ${\bar{\varphi }}^{T}$ can exceed *F*_ST_ under the coancestry model (where ${\bar{\theta }}^{T}\leq {\bar{\varphi }}^{T}$) and also under extreme local kinship, where ${\hat{F}}_{\text{ST}}^{\text{std}}$ in Eq (25) converges to a negative value.

#### Adjusted consistent oracle *F*_ST_ estimators and the “bias coefficient”

Here we explore two adjustments to ${\hat{F}}_{\text{ST}}^{\text{std}}$ from IAFs in Eq (26) that rely on having minimal additional information needed to correct its bias. These “oracle” approaches require information that is not known in practice, but this exercise helps us understand the problem more deeply and finds further connections between the various *F*_ST_ estimators.

If ${\bar{\theta }}^{T}$ is known, the bias in Eq (26) can be reversed, yielding the consistent estimator

$$\begin{array}{c} {\hat{F}}_{\text{ST}}^{\prime }={\hat{F}}_{\text{ST}}^{\text{std}}\left(1-{\bar{\theta }}^{T}\right)+{\bar{\theta }}^{T}\overset{\text{a.s.}}{\underset{m\to \infty }{\to }}F_{\text{ST}}. \end{array}$$ (27)

Consistent estimates are also possible if a scaled version of ${\bar{\theta }}^{T}$ is known, namely

$$\begin{array}{c} s^{T}=\frac{{\bar{\theta }}^{T}}{F_{\text{ST}}}=\frac{\sum_{j=1}^{n}\sum_{k=1}^{n}w_{j}w_{k}\theta_{jk}^{T}}{\sum_{j=1}^{n}w_{j}\theta_{jj}^{T}}, \end{array}$$ (28)

which we call the “bias coefficient” and which has interesting properties. The bias coefficient quantifies the departure from the independent subpopulations model by comparing the mean coancestry ($\theta_{jk}^{T}$) to the mean inbreeding coefficient ($\theta_{jj}^{T}$), and given *F*_ST_ > 0 satisfies 0 < *s*^*T*^ ≤ 1 (S1 Text). The limit in Eq (26) in terms of *s*^*T*^ is

$$\begin{array}{c} {\hat{F}}_{\text{ST}}^{\text{std}}\overset{\text{a.s.}}{\underset{m\to \infty }{\to }}F_{\text{ST}}\frac{1-s^{T}}{1-s^{T}F_{\text{ST}}}. \end{array}$$ (29)

Treating the limit as equality and solving for *F*_ST_ yields the following consistent estimator:

$${\hat{\sigma }}_{i}^{2}=\frac{1}{1-s^{T}}\sum_{j=1}^{n}w_{j}{\left(\pi_{ij}-{\hat{p}}_{i}^{T}\right)}^{2},$$ (30)

$${\hat{F}}_{\text{ST}}^{\prime \prime }=\frac{{\hat{F}}_{\text{ST}}^{\text{std}}}{1-s^{T}\left(1-{\hat{F}}_{\text{ST}}^{\text{std}}\right)}=\frac{\sum_{i=1}^{m}{\hat{\sigma }}_{i}^{2}}{\sum_{i=1}^{m}{\hat{p}}_{i}^{T}(1-{\hat{p}}_{i}^{T})+s^{T}{\hat{\sigma }}_{i}^{2}}\overset{\text{a.s.}}{\underset{m\to \infty }{\to }}F_{\text{ST}}.$$ (31)

Note that ${\hat{\sigma }}_{i}^{2}$ and ${\hat{F}}_{\text{ST}}^{\text{indep}}$ from Eqs (12) and (13) are the special case of Eqs (30) and (31) for uniform weights and $s^{T}=\frac{1}{n}$; hence, ${\hat{F}}_{\text{ST}}^{\prime \prime }$ generalizes ${\hat{F}}_{\text{ST}}^{\text{indep}}$.

Lastly, using either Eqs (26) or (29), the relative error of ${\hat{F}}_{\text{ST}}^{\text{std}}$ converges to

$$\begin{array}{c} 1-\frac{{\hat{F}}_{\text{ST}}^{\text{std}}}{F_{\text{ST}}}\overset{\text{a.s.}}{\underset{m\to \infty }{\to }}\frac{{\bar{\theta }}^{T}(1-F_{\text{ST}})}{F_{\text{ST}}(1-{\bar{\theta }}^{T})}=s^{T}\frac{1-F_{\text{ST}}}{1-s^{T}F_{\text{ST}}}, \end{array}$$ (32)

which is approximated by *s*^*T*^ if *F*_ST_ ≪ 1, hence the name “bias coefficient”. Note *s*^*T*^ varies depending on the choice of *T*, which is necessary since *F*_ST_ (and hence the relative bias of ${\hat{F}}_{\text{ST}}^{\text{std}}$ from *F*_ST_) depends on the choice of *T*.

### A new approach for kinship and *F*_ST_ estimation

Here, we propose a new estimation approach for kinship coefficients that has properties favorable for obtaining nearly unbiased estimates (panel “New Kinship Estimator” in Fig 1). These new kinship estimates yield an improved *F*_ST_ estimator. We present the general approach and implement a simple version of one key estimator that results in the complete proof-of-principle estimator that is evaluated in the next section and applied to human data in [59]. We also compare our approach to a related estimator of non-IBD linearly-transformed kinship values [20–22] that was proposed concurrently to ours [58].

#### General approach

In this subsection we develop our new estimator in two steps. First, we compute a new statistic *A*_*jk*_ that is proportional in the limit of infinite loci to $\varphi_{jk}^{T}-1$ times a nuisance factor *v*^*T*^. Second, we estimate and remove *v*^*T*^ to yield the proposed estimator ${\hat{\varphi }}_{jk}^{T,\text{new}}$. ${\hat{A}}_{\text{min}}$—an estimator of the limit of the minimum *A*_*jk*_—yields *v*^*T*^ if the least related pair of individuals in the data has $\varphi_{jk}^{T}=0$, which sets *T* to the MRCA population of all the individuals in the data. The new kinship estimator immediately results in new inbreeding (${\hat{f}}_{j}^{T,\text{new}}$) and *F*_ST_ (${\hat{F}}_{\text{ST}}^{\text{new}}$) estimators. This general approach leaves the implementation of ${\hat{A}}_{\text{min}}$ open; the simple implementation applied in this work is described in subsection **Proof-of-principle kinship estimator using subpopulation labels**, but our method can be readily improved by substituting in a better ${\hat{A}}_{\text{min}}$ in the future.

Applying the method of moments to Eq (5), we derive the following statistic (S1 Text), whose expectation is proportional to $\varphi_{jk}^{T}-1$:

$$\begin{array}{c} \begin{array}{cc} A_{jk} & =\frac{1}{m}\sum_{i=1}^{m}\left(x_{ij}-1\right)\left(x_{ik}-1\right)-1,\\ E[A_{jk}|T] & =(\varphi_{jk}^{T}-1)v_{m}^{T},\;\text{where}\\ v_{m}^{T} & =\frac{4}{m}\sum_{i=1}^{m}p_{i}^{T}(1-p_{i}^{T}). \end{array} \end{array}$$ (33)

Compared to the standard kinship estimator in Eq (19), which has a complex asymptotic bias determined by *n* parameters (${\bar{\varphi }}_{j}^{T}$ for each *j* ∈ {1, …, *n*}), the *A*_*jk*_ statistics estimate kinship with a bias controlled by the sole unknown parameter $v_{m}^{T}$ shared by all pairs of individuals. The key to estimating $v_{m}^{T}$ is to notice that if $\varphi_{jk}^{T}=0$ then $E\left[A_{jk}|T\right]=-v_{m}^{T}$. Thus, assuming $\min_{j,k}\varphi_{jk}^{T}=0$, which sets *T* to the MRCA population, then the minimum *A*_*jk*_ yields the nuisance parameter. However, we recommend using a more stable estimate than the minimum *A*_*jk*_ to unbias all *A*_*jk*_, such as the estimator presented in the next subsection.

In general, suppose ${\hat{A}}_{\text{min}}$ is a consistent estimator of the limit of the minimum *E*[*A*_*jk*_|*T*], or equivalently,

$$\begin{array}{c} {\hat{A}}_{\text{min}}\overset{\text{a.s.}}{\underset{m\to \infty }{\to }}-v^{T}, \end{array}$$

along with the assumption that $v_{m}^{T}\underset{m\to \infty }{\to }v^{T}$ for some *v*^*T*^ ≠ 0. Our new kinship estimator follows directly from replacing $v_{m}^{T}$ with $-{\hat{A}}_{\text{min}}$ and solving for $\varphi_{jk}^{T}$ in Eq (33), which results in a consistent kinship estimator (given the convergence proof of section **Assessing the accuracy of genome-wide ratio estimators**):

$$\begin{array}{c} {\hat{\varphi }}_{jk}^{T,\text{new}}=1-\frac{A_{jk}}{{\hat{A}}_{\text{min}}}\overset{\text{a.s.}}{\underset{m\to \infty }{\to }}\varphi_{jk}^{T}. \end{array}$$ (34)

The resulting new inbreeding coefficient estimator is

$$\begin{array}{c} {\hat{f}}_{j}^{T,\text{new}}=2{\hat{\varphi }}_{jj}^{T,\text{new}}-1\overset{\text{a.s.}}{\underset{m\to \infty }{\to }}f_{j}^{T}, \end{array}$$ (35)

and the new *F*_ST_ estimator is consistent for locally-outbred individuals (estimates Eq (4)):

$$\begin{array}{c} {\hat{F}}_{\text{ST}}^{\text{new}}=\sum_{j=1}^{n}w_{j}{\hat{f}}_{j}^{T,\text{new}}\overset{\text{a.s.}}{\underset{m\to \infty }{\to }}F_{\text{ST}}. \end{array}$$ (36)

Thus, only the implementation of ${\hat{A}}_{\text{min}}$ is left unspecified from this general estimation approach of kinship and *F*_ST_. The implementation of ${\hat{A}}_{\text{min}}$ used in the analyses in this work is given in the next subsection.

#### Proof-of-principle kinship estimator using subpopulation labels

To showcase the potential of the new estimators, we implement a simple proof-of-principle version of ${\hat{A}}_{\text{min}}$ needed for our new kinship estimator (${\hat{\varphi }}_{jk}^{T,\text{new}}$ in Eq (34)). This ${\hat{A}}_{\text{min}}$ relies on an appropriate partition of the *n* individuals into *K* subpopulations (denoted *S*_*u*_ for *u* ∈ {1, …, *K*}), where the only requirement is that the kinship coefficients between pairs of individuals across the two most unrelated subpopulations is zero, as detailed below. Note that, unlike the the independent subpopulations model of section ***F*_ST_ estimation based on the independent subpopulations model**, these *K* subpopulations need not be independent nor unstructured. The desired estimator ${\hat{A}}_{\text{min}}$ is the minimum average *A*_*jk*_ over all subpopulation pairs:

$$\begin{array}{c} {\hat{A}}_{\text{min}}=\min_{u\neq v}\frac{1}{|S_{u}\left|\right|S_{v}|}\sum_{j\in S_{u}}\sum_{k\in S_{v}}A_{jk}. \end{array}$$ (37)

This ${\hat{A}}_{\text{min}}$ consistently estimates the limit of the minimum *A*_*jk*_ if $\varphi_{jk}^{T}=0\;\forall j\in S_{u},\forall k\in S_{v}$ for the least related pair of subpopulations *S*_*u*_, *S*_*v*_.

This estimator should work well for individuals truly divided into subpopulations, but may be biased for a poor choice of subpopulations, in particular if the minimum mean $\varphi_{jk}^{T}$ between subpopulations is far greater than zero. For this reason, inspection of the kinship estimates is required and careful construction of appropriate subpopulations may be needed. See our analysis of human data for detailed examples [59]. Future work could focus on a more general ${\hat{A}}_{\text{min}}$ that circumvents the need for subpopulations of our proof-of-principle estimator.

#### Comparison to the Weir-Goudet kinship estimator for individuals

Here we analyze the Weir-Goudet (WG) kinship estimator for individuals [20–22]. This has connections to our new estimator but differs in having the goal of estimating linearly-transformed kinship values. In our framework, the WG estimator is given by

$$\begin{array}{c} {\hat{\varphi }}_{jk}^{T,\text{WG}}=1-\frac{A_{jk}}{{\hat{A}}_{\text{avg}}},\;\text{where}\;{\hat{A}}_{\text{avg}}=\frac{2}{n(n-1)}\sum_{j=2}^{n}\sum_{k=1}^{j-1}A_{jk}. \end{array}$$

Therefore, this estimator differs from our proposal [58] by replacing our ${\hat{A}}_{\text{min}}$ with ${\hat{A}}_{\text{avg}}$. Under the kinship model, the expectation of ${\hat{A}}_{\text{avg}}$ is

$$\begin{array}{c} E[{\hat{A}}_{\text{avg}}|T]=({\tilde{\varphi }}^{T}-1)v_{m}^{T},\;\text{where}\;{\tilde{\varphi }}^{T}=\frac{2}{n(n-1)}\sum_{j=2}^{n}\sum_{k=1}^{j-1}\varphi_{jk}^{T}. \end{array}$$

Therefore, the limit of this estimator is

$${\hat{\varphi }}_{jk}^{T,\text{WG}}\overset{\text{a.s.}}{\underset{m\to \infty }{\to }}\frac{\varphi_{jk}^{T}-{\tilde{\varphi }}^{T}}{1-{\tilde{\varphi }}^{T}},$$ (38)

which agrees with calculations in the original WG work [20–22]. Note that, assuming that kinship coefficients must be non-negative, the above estimator recovers the kinship IBD probabilities if and only if ${\tilde{\varphi }}^{T}=0$ which holds if and only if $\varphi_{jk}^{T}=0$ for every pair of individuals *j* ≠ *k*. The resulting WG inbreeding coefficient estimator is

$${\hat{f}}_{jk}^{T,\text{WG}}=2{\hat{\varphi }}_{jk}^{T,\text{WG}}-1\overset{\text{a.s.}}{\underset{m\to \infty }{\to }}\frac{f_{j}^{T}-{\tilde{\varphi }}^{T}}{1-{\tilde{\varphi }}^{T}},$$

which estimates linearly-transformed inbreeding values [21]. Therefore, the resulting WG *F*_ST_ estimator (for individuals) also targets a linearly-transformed *F*_ST_ value (under locally-outbred individuals, where *F*_ST_ is given by Eq (4)), namely

$${\hat{F}}_{\text{ST}}^{\text{WG}}=\frac{1}{n}\sum_{j=1}^{n}{\hat{f}}_{j}^{T,\text{WG}}\overset{\text{a.s.}}{\underset{m\to \infty }{\to }}\frac{F_{\text{ST}}-{\tilde{\varphi }}^{T}}{1-{\tilde{\varphi }}^{T}}.$$

The WG authors also briefly consider a variant of their kinship estimator that is normalized using the minimum kinship value as we did, developed concurrently with our approach [58], but was largely dismissed as an unnecessary correction [21, 76]. See S1 Text for a detailed proof that the general estimator framework we propose here (Eqs (33) and (34)) is algebraically equivalent to our original formulation in [58].

Note that the original WG does not estimate *F*_ST_ from individuals as considered above; instead, *F*_ST_ is estimated from coancestry estimates for subpopulations (which equals our HudsonK for biallelic loci, S1 Text) [20–22]. For completeness, we consider both kinds of *F*_ST_ estimates in the evaluations that follow.

### Simulations evaluating *F*_ST_ and kinship estimators

#### Overview of simulations

**Fig 2:**
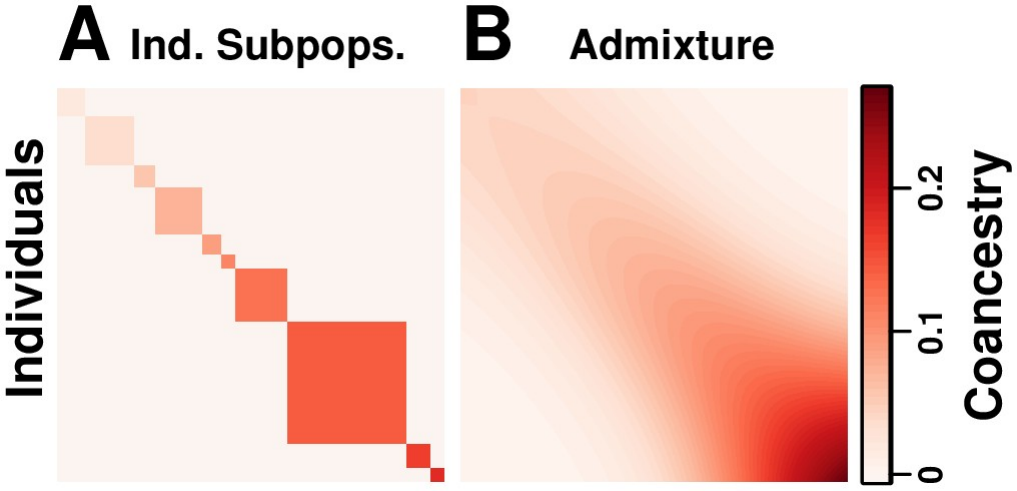
Coancestry matrices of simulations. Both panels have *n* = 1000 individuals along both axes, *K* = 10 subpopulations (final or intermediate), and *F*_ST_ = 0.1. Color corresponds to $\theta_{jk}^{T}$ between individuals *j* and *k* (equal to $\varphi_{jk}^{T}$ off-diagonal, $f_{j}^{T}$ along the diagonal). (A) The independent subpopulations model has $\theta_{jk}^{T}=0$ between subpopulations, and varying $\theta_{jj}^{T}$ per subpopulation, resulting in a block-diagonal coancestry matrix. (B) Our admixture scenario models a 1D geography with extensive admixture and intermediate subpopulation differentiation that increases with distance, resulting in a smooth coancestry matrix with no independent subpopulations (no $\theta_{jk}^{T}=0$ between blocks). Individuals are ordered along each axis by geographical position.

**Fig 3:**
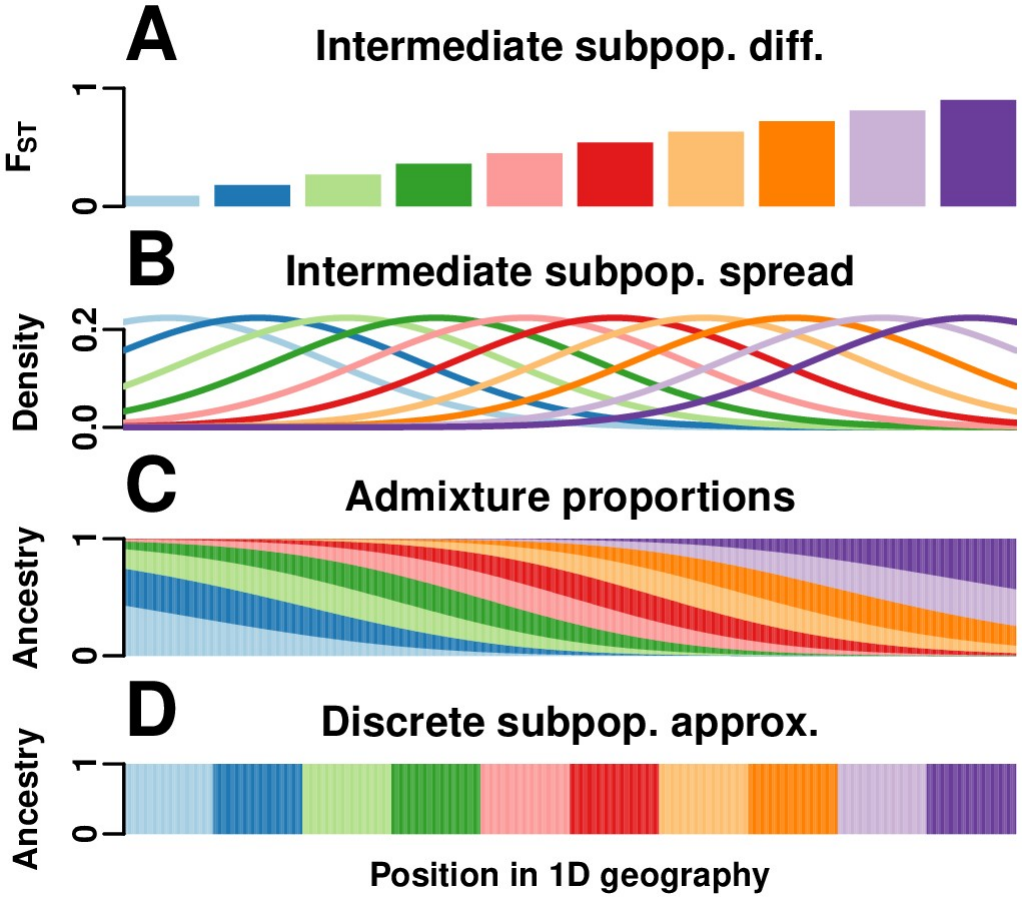
1D admixture scenario. We model a 1D geography population that departs strongly from the independent subpopulations model. (A) *K* = 10 intermediate subpopulations, evenly spaced on a line, evolved independently in the past with *F*_ST_ increasing with distance, which models a sequence of increasing founder effects (from left to right) to mimic the global human population. (B) Once differentiated, individuals in these intermediate subpopulations spread by random walk modeled by Normal densities. (C) *n* = 1000 individuals, sampled evenly in the same geographical range, are admixed proportionally to the previous Normal densities. Thus, each individual draws most of its alleles from the closest intermediate subpopulation, and draws the fewest alleles from the most distant populations. Long-distance random walks of intermediate subpopulation individuals results in kinship for admixed individuals that decays smoothly with distance in Fig 2B. (D) For *F*_ST_ estimators that require a partition of individuals into subpopulations, individuals are clustered by geographical position (*K* = 10).

We simulate genotypes from two models to illustrate our results when the true population structure parameters are known. Both simulations have clearly-defined IBD probability parameters in terms of the MRCA population. The first simulation satisfies the independent subpopulations model that existing *F*_ST_ estimators assume. The second simulation is from an admixture model with no independent subpopulations and pervasive kinship designed to induce large downward biases in existing kinship and *F*_ST_ estimators (Fig 2). This admixture scenario resembles the population structure we estimated for Hispanics in the 1000 Genomes Project [59]: compare the simulated kinship matrix (Fig 2B) and admixture proportions (Fig 3C) to our estimates on the real data [59]. Both simulations have *n* = 1000 individuals, *m* = 300, 000 loci, and *K* = 10 subpopulations or intermediate subpopulations. These simulations have *F*_ST_ = 0.1, comparable to previous estimates between human populations (in 1000 Genomes, the estimated *F*_ST_ between CEU (European-Americans) and CHB (Chinese) is 0.106, between CEU and YRI (Yoruba from Nigeria) it is 0.139, and between CHB and YRI it is 0.161 [23]).

The independent subpopulations simulation satisfies the HudsonK and BayeScan estimator assumptions: each independent subpopulation *S*_*u*_ has a different *F*_ST_ value of $f_{S_{u}}^{T}$ relative to the MRCA population *T* (Fig 2A). Ancestral allele frequencies $p_{i}^{T}$ are drawn uniformly between 0.01 and 0.5. Allele frequencies $p_{i}^{S_{u}}$ for *S*_*u*_ and locus *i* are drawn independently from the Balding-Nichols (BN) distribution [3] with parameters $p_{i}^{T}$ and $f_{S_{u}}^{T}$. Every individual *j* in subpopulation *S*_*u*_ draws alleles randomly with probability $p_{i}^{S_{u}}$. Subpopulation sample sizes are drawn randomly (Methods, section **Simulations**).

The admixture simulation corresponds to a “BN-PSD” model [6, 27, 34, 60, 77]: the intermediate subpopulations are independent subpopulations that draw $p_{i}^{S_{u}}$ from the BN model, then each individual *j* constructs its allele frequencies as $\pi_{ij}=\sum_{u=1}^{K}p_{i}^{S_{u}}q_{ju}$, which is a weighted average of the subpopulation allele frequencies $p_{i}^{S_{u}}$ with the admixture proportions *q*_*ju*_ of individual *j* and subpopulation *u* as weights (which satisfy $\sum_{u=1}^{K}q_{ju}=1$), as in the Pritchard-Stephens-Donnelly (PSD) admixture model [63–65]. We constructed *q*_*ju*_ that model admixture resulting from spread by random walk of the intermediate subpopulations along a one-dimensional geography, as follows. Intermediate subpopulations *S*_*u*_ are placed on a line with differentiation $f_{S_{u}}^{T}$ that grows with distance, which corresponds to a serial founder effect (Fig 3A). Upon differentiation, individuals in each *S*_*u*_ spread by random walk, a process modeled by Normal densities (Fig 3B). Admixed individuals derive their ancestry proportional from these Normal densities, resulting in a genetic structure governed by geography (Figs 3C and 2B) and departing strongly from the independent subpopulations model (Fig 3D). The amount of spread—which sets the mean kinship across all individuals—was chosen to give a bias coefficient of $s^{T}=\frac{{\bar{\theta }}^{T}}{F_{\text{ST}}}=0.5$, which by Eq (32) results in a large downward bias for ${\hat{F}}_{\text{ST}}^{\text{std}}$ (in contrast, the independent subpopulations simulation has *s*^*T*^ = 0.1). The true coancestry and *F*_ST_ parameters of this simulation are given by the $f_{S_{u}}^{T}$ values of the intermediate subpopulations and the admixture coefficients *q*_*ju*_ of the individuals via the following equations [57]:

$$\begin{array}{c} \begin{array}{cc} \theta_{jk}^{T} & =\sum_{u=1}^{K}q_{ju}q_{ku}f_{S_{u}}^{T},\\ F_{\text{ST}} & =\sum_{j=1}^{n}\sum_{u=1}^{K}w_{j}q_{ju}^{2}f_{S_{u}}^{T}. \end{array} \end{array}$$ (39)

The first equation above connecting coancestry to admixture proportions was derived independently in other work [62], but the *F*_ST_ for the admixed individuals was absent and instead follows from our generalized *F*_ST_ definition given in Eq (9). See Methods, section **Simulations** for additional details regarding these simulations.

#### Evaluation of *F*_ST_ estimators

Our admixture simulation illustrates the large biases that can arise if existing *F*_ST_ estimators that require independent subpopulations or *F*_ST_ estimates derived from existing kinship estimators are misapplied to arbitrary population structures to estimate the generalized *F*_ST_, and demonstrate the higher accuracy of our new *F*_ST_ estimator (${\hat{F}}_{\text{ST}}^{\text{new}}$ given by the combination of Eqs (36) and (37)). The WC *F*_IT_ (total inbreeding) estimator was also evaluated.

**Fig 4:**
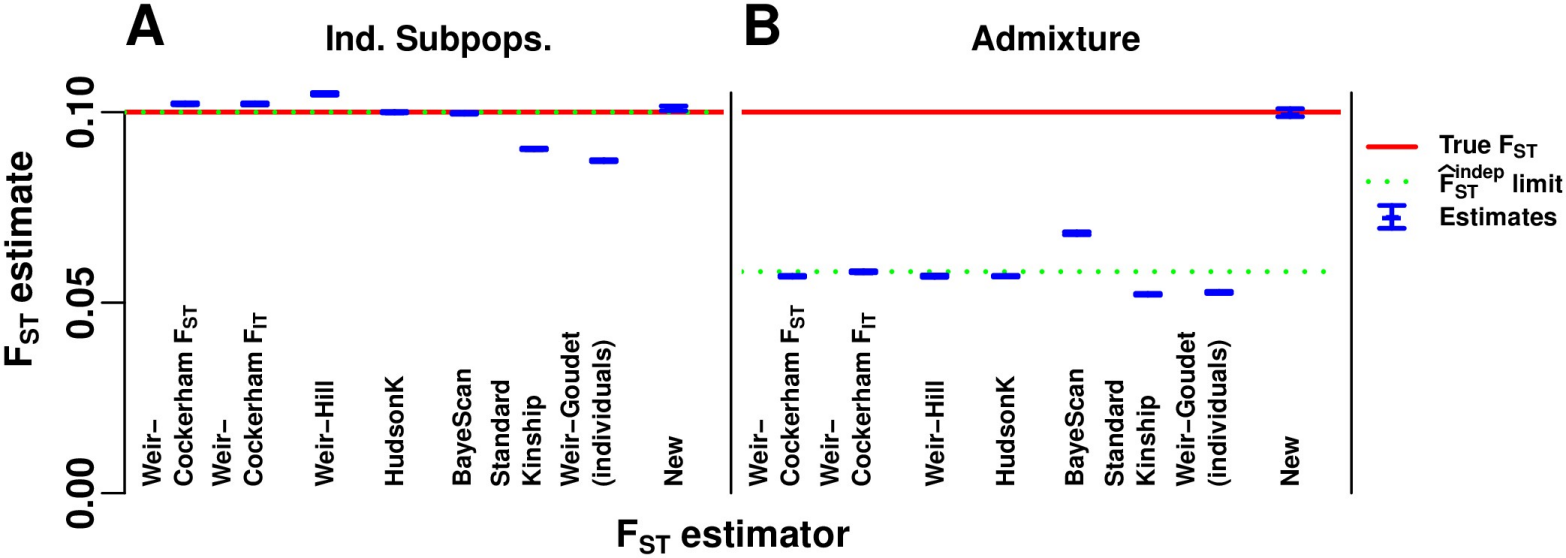
Evaluation of *F*_ST_ estimators. The Weir-Cockerham, Weir-Hill, Weir-Goudet (for individuals), HudsonK (equal to Weir-Goudet for subpopulations, S1 Text), BayeScan, ${\hat{F}}_{\text{ST}}^{\text{std}}$ in Eq (25) derived from the standard kinship estimator, and our new *F*_ST_ estimator in Eqs (34) and (37), are evaluated on simulated genotypes from our two models (Fig 2). The Weir-Cockerham *F*_IT_ estimator was also included to show that estimation of total inbreeding behaves similarly to *F*_ST_ estimators. (A) The independent subpopulations model required by the Weir-Hill, HudsonK, and BayeScan *F*_ST_ estimators. All but standard kinship (${\hat{F}}_{\text{ST}}^{\text{std}}$) and Weir-Goudet (for individuals) recover the target *F*_ST_ IBD probability in Eq (9) (red line) with small errors. (B) Our admixture scenario, which has no independent subpopulations, was constructed so ${\hat{F}}_{\text{ST}}^{\text{std}}\approx \frac{1}{2}F_{\text{ST}}$. Only our new estimates are accurate. The rest of these estimators give values smaller than the target *F*_ST_ IBD probability, which result from treating kinship as zero between every subpopulations imposed by geographic clustering (or between individuals for Standard Kinship and Weir-Goudet). The ${\hat{F}}_{\text{ST}}^{\text{indep}}$ estimator limit in Eq (14) (green dotted line) overlaps the true *F*_ST_ (red line) in (A) but not (B). Estimates (blue) include 95% prediction intervals (often too narrow to see) from 39 independently-simulated genotype matrices for each model (Methods, section **Prediction intervals**).

First, we test these estimators in our independent subpopulations simulation. The HudsonK (Methods, section **Generalized HudsonK *F*_ST_ estimator**) and BayeScan *F*_ST_ estimators are consistent in this simulation, since their assumptions are satisfied (Fig 4A). The WC *F*_ST_ estimator assumes that $f_{S_{u}}^{T}=F_{\text{ST}}$ for all subpopulations *S*_*u*_, which does not hold; nevertheless, WC has only a small bias (Fig 4A). The WC *F*_IT_ estimator arrives at similar estimates, as it should since there is no local inbreeding, so the true *F*_IT_ also equals *F*_ST_. The Weir-Hill estimator permits different $f_{S_{u}}^{T}$ values per subpopulation, but assigns equal weight to individuals rather than subpopulations (Methods, section **The Weir-Hill *F*_ST_ estimator**), resulting in a slightly different target *F*_ST_ (we verified that these estimates are unbiased for this *F*_ST_). For comparison, we show the standard kinship-based ${\hat{F}}_{\text{ST}}^{\text{std}}$ in Eq (25) (weights from Methods, section **Simulations**) and ${\hat{F}}_{\text{ST}}^{\text{WG}}$ based on the Weir-Goudet kinship estimates for individuals, both of which do not have corrections that would make them consistent under the independent subpopulations model. Since the number of subpopulations *K* is large, ${\hat{F}}_{\text{ST}}^{\text{std}}$ has a small relative bias of about $s^{T}=\frac{1}{K}=10\%$ (Fig 4A); greater bias is expected for smaller *K*. Our new *F*_ST_ estimator has a very small bias in this simulation resulting from estimating the minimum kinship from the smallest kinship between subpopulations (see Eq (37)) rather than their average as HudsonK does implicitly (Fig 4A).

Next we test these estimators in our admixture simulation. To apply the *F*_ST_ estimators that require subpopulations to the admixture model, individuals are clustered into subpopulations by their geographical position (Fig 3D). We find that estimates of all existing methods are smaller than the true *F*_ST_ by nearly half, as predicted by the limit of ${\hat{F}}_{\text{ST}}^{\text{indep}}$ in Eq (14) (Fig 4B). The WC *F*_IT_ estimator obtains slightly larger estimates than the WC *F*_ST_ estimator, but overall remains as biased as the other *F*_ST_ estimators, showing that the use of a total inbreeding estimator for independent subpopulations displays the same bias as the corresponding *F*_ST_ estimator. By construction, the kinship-based ${\hat{F}}_{\text{ST}}^{\text{std}}$ also has a large relative bias of about *s*^*T*^ = 50%; remarkably, all existing *F*_ST_ estimators for subpopulations suffer from comparable biases. Thus, the corrections for independent subpopulations present in the WC, Weir-Hill and HudsonK estimators, or the Bayesian likelihood modeling of BayeScan, are insufficient for accurate estimation of the target generalized *F*_ST_ (Eq (9)) in this admixture scenario. Only our new *F*_ST_ estimator achieves accurate estimates of the generalized *F*_ST_ in the admixture simulation (Fig 4B).

#### Evaluation of kinship estimators

**Fig 5:**
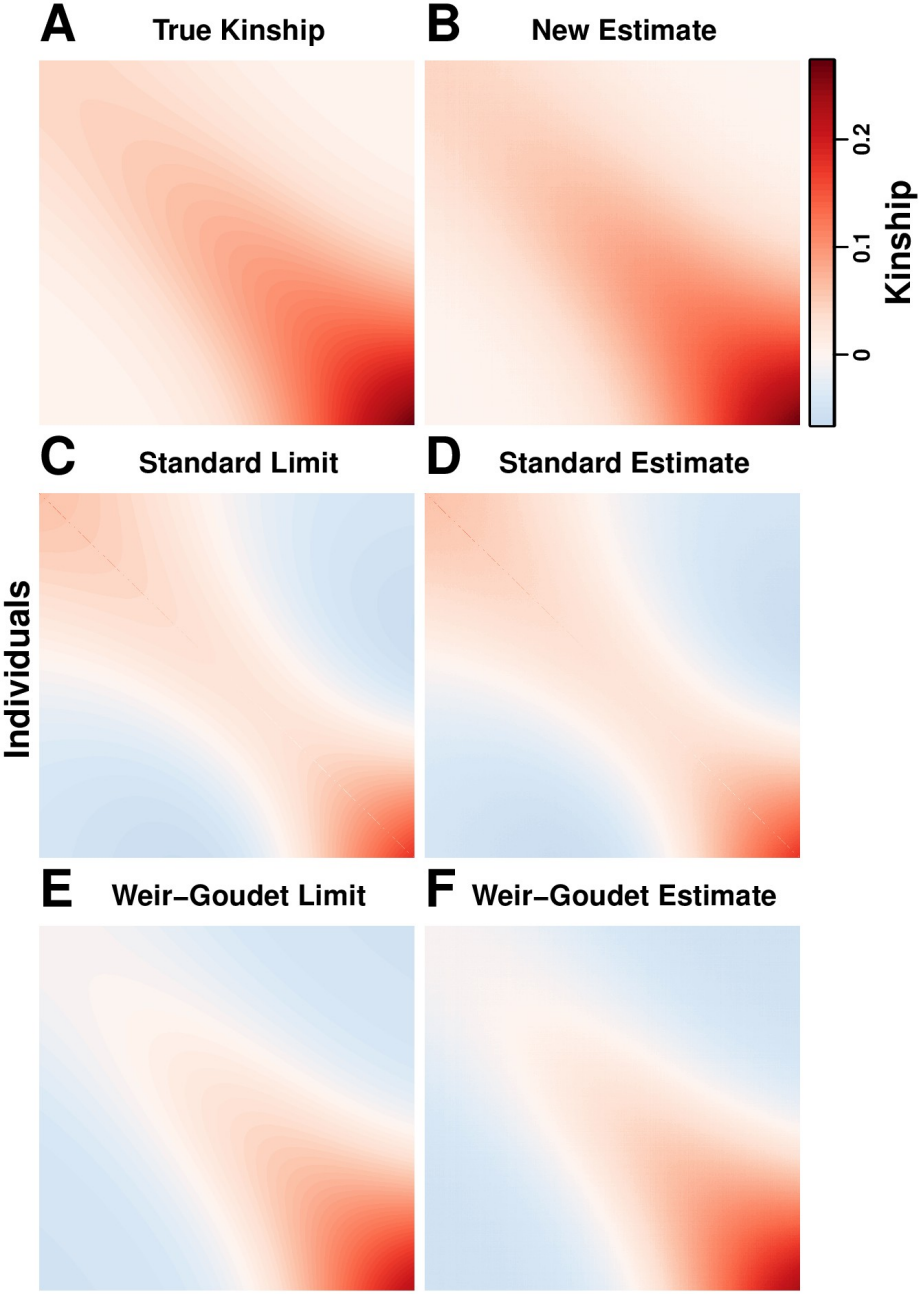
Evaluation of kinship estimators. Observed accuracy for two existing kinship coefficient estimators is illustrated in our admixture simulation and contrasted to the nearly unbiased estimates of our new estimator. Plots show *n* = 1000 individuals along both axes, and color corresponds to $\varphi_{jk}^{T}$ between individuals *j* ≠ *k* and to $f_{j}^{T}$ along the diagonal ($f_{j}^{T}$ is in the same scale as $\varphi_{jk}^{T}$ for *j* ≠ *k*; plotting $\varphi_{jj}^{T}$, which have a minimum value of $\frac{1}{2}$, would result in a discontinuity in this figure). (A) True kinship matrix. (B) Estimated kinship using our new estimator in Eqs (34) and (37) from simulated genotypes recovers the true kinship matrix with high accuracy. (C) Theoretical limit of ${\hat{\varphi }}_{jk}^{T,\text{std}}$ in Eq (19) as the number of independent loci goes to infinity demonstrates the accuracy of our bias predictions under the kinship model. (D) Standard kinship estimates ${\hat{\varphi }}_{jk}^{T,\text{std}}$ given by Eq (18) from simulated genotypes are downwardly biased on average and distorted by pair-specific amounts. (E) Theoretical limit of the Weir-Goudet kinship estimator given by Eq (38). (F) Weir-Goudet kinship estimates from the same simulated genotypes agree with our calculated limit.

**Fig 6:**
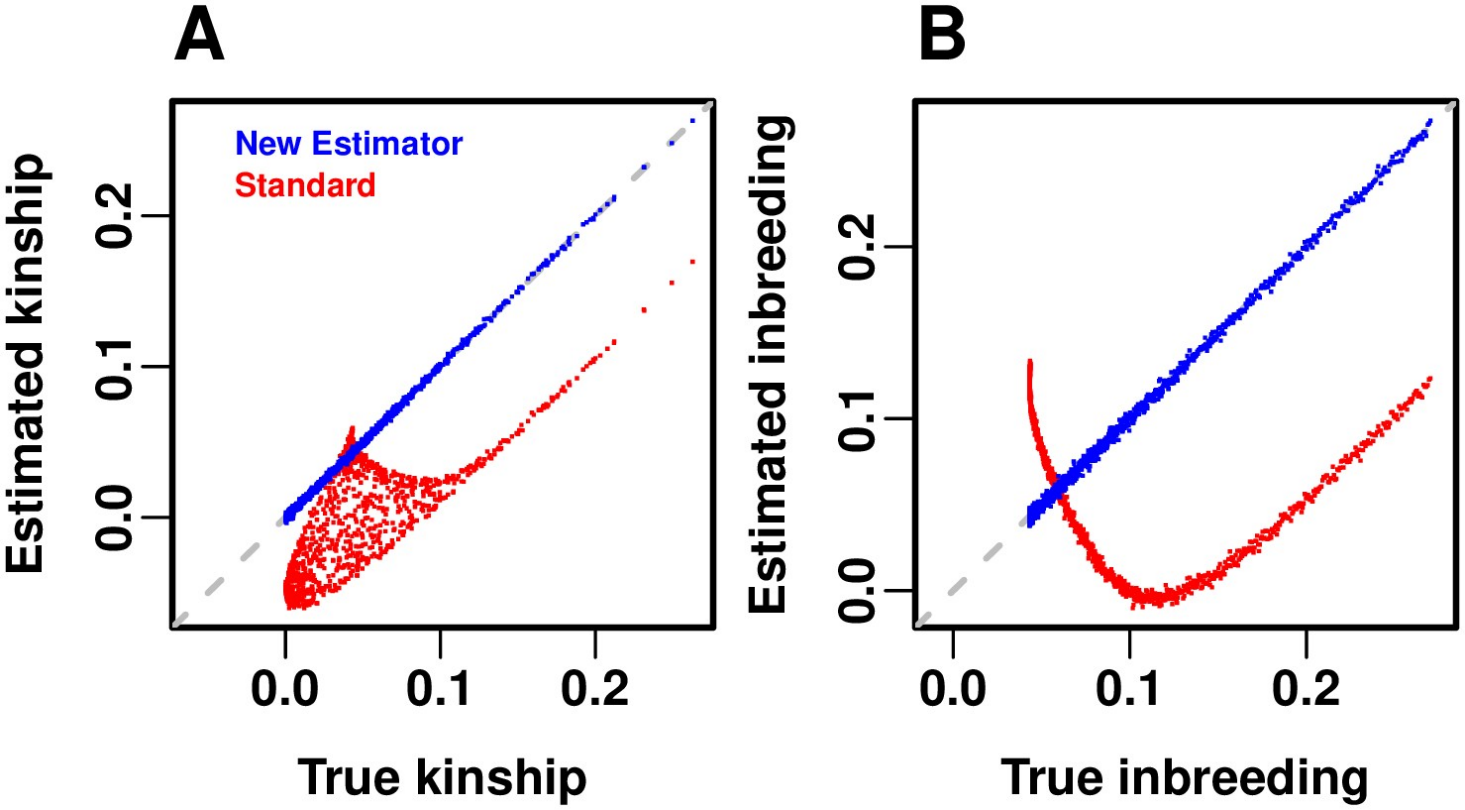
Accuracy of kinship estimators. Here the estimated kinship values are directly compared to their true values, in the same admixture simulation data (*n* = 1000 individuals) shown in the previous figure. (A) Kinship between different individuals (excluding inbreeding). The new estimator has practically no bias in this evaluation (falls on the 1-1 dashed gray line). The standard estimator has a complex, non-linear bias that covers a large area of errors. (B) Inbreeding comparison, shows the bias of the standard estimate follows a different pattern for inbreeding compared to kinship between individuals. To better visualize and compare data across panels, a random subset of *n* points (out of the original *n*(*n* − 1)/2 unique individual pairs) were plotted in (A), matching the number of individuals (number of points in (B)).

Our admixture simulation illustrates the distortions of the standard kinship estimator ${\hat{\varphi }}_{jk}^{T,\text{std}}$ in Eq (18), the linearly-transformed kinship values given by the Weir-Goudet estimator, and demonstrates the improved accuracy of our new kinship estimator ${\hat{\varphi }}_{jk}^{T,\text{new}}$ given by the combination of Eqs (34) and (37). Kinship matrix estimates and their limits are visualized as heatmaps in Fig 5, whereas estimator accuracy is shown directly in Fig 6. The limit of the standard estimator ${\hat{\varphi }}_{jk}^{T,\text{std}}$ in Eq (18) would have had a uniform bias if ${\bar{\varphi }}_{j}^{T}={\bar{\varphi }}^{T}$ held for all individuals *j*. For that reason, our admixture simulation has varying differentiation $f_{S_{u}}^{T}$ per intermediate subpopulation *S*_*u*_ (Fig 3A), which causes large differences in ${\bar{\varphi }}_{j}^{T}$ per individual *j* and therefore large distortions in ${\hat{\varphi }}_{jk}^{T,\text{std}}$. The Weir-Goudet approach estimates the linearly-transformed kinship values calculated in Eq (38).

Our new kinship estimator (Fig 5B) recovers the true kinship matrix of this complex population structure (Fig 5A), with an RMSE of 2.83% relative to the mean $\varphi_{jk}^{T}$ (Fig 6). In contrast, estimates using the standard estimator have a large overall downward bias (Fig 5C), resulting in an RMSE of 115.72% from the true $\varphi_{jk}^{T}$ relative to the mean $\varphi_{jk}^{T}$ (Fig 6). Additionally, estimates from ${\hat{\varphi }}_{jk}^{T,\text{std}}$ are very distorted, with an abundance of ${\hat{\varphi }}_{jk}^{T,\text{std}}<\varphi_{jk}^{T}$ cases—some of which are negative estimates (blue in Fig 5C)—but remarkably also cases with ${\hat{\varphi }}_{jk}^{T,\text{std}}>\varphi_{jk}^{T}$ (top left corner of Figs 5C and 6).

Now we compare the convergence of the ratio-of-means and mean-of-ratios versions of the standard kinship estimator to their biased limit we calculated in Eq (19) (Fig 5D). The ratio-of-means estimate ${\hat{\varphi }}_{jk}^{T,\text{std}}$ (Fig 5C) has an RMSE of 2.14% from its limit relative to the mean $\varphi_{jk}^{T}$. In contrast, the mean-of-ratios estimates that are prevalent in the literature have a greater RMSE of 10.77% from the same limit in Eq (19). Thus, as expected from our theoretical results in section **Assessing the accuracy of genome-wide ratio estimators**, the ratio-of-means estimate is much closer to the desired limit than the mean-of-ratio estimate. The distortions are similar for the estimator that uses IAFs in Eq (24), with reduced RMSEs from its limit of 0.32% and 8.82% for the ratio-of-means and mean-of-ratios estimates, respectively.

#### Evaluation of oracle-adjusted *F*_ST_ estimators

**Fig 7:**
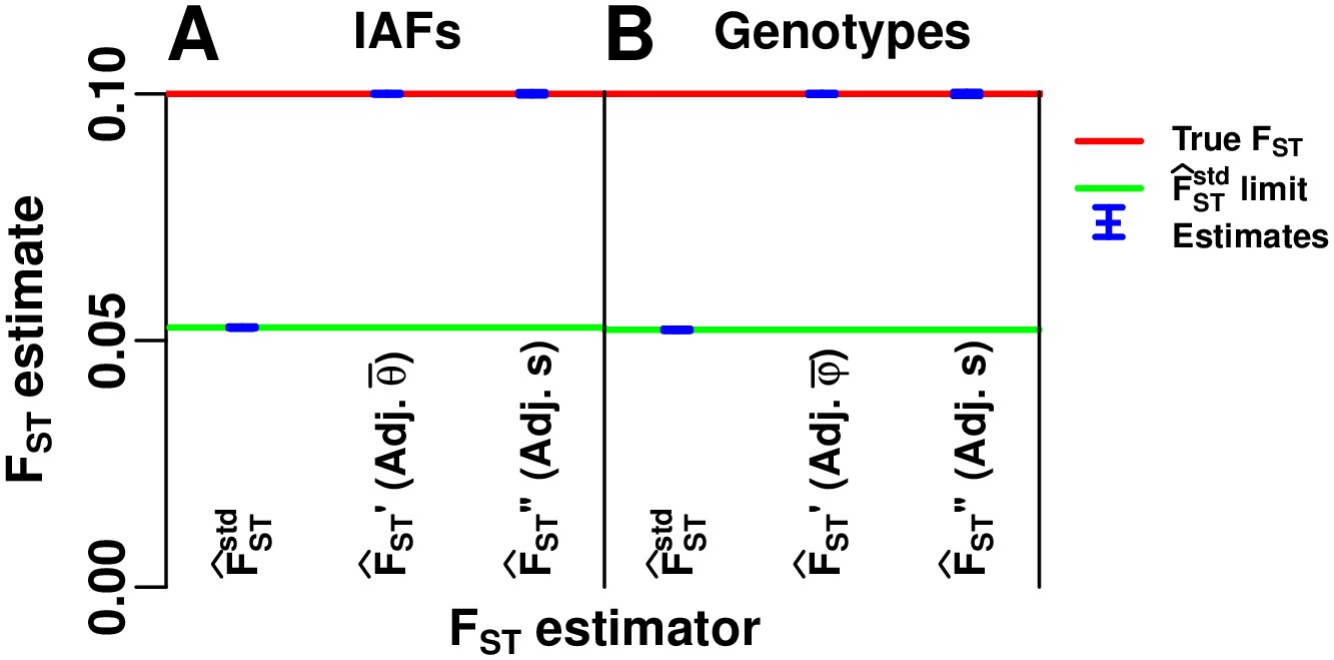
Evaluation of standard and adjusted *F*_ST_ estimators. The convergence values we calculated for the standard kinship plug-in and adjusted *F*_ST_ estimators are validated using our admixture simulation. All adjusted estimators are unbiased but are “oracle” methods, since the mean kinship (${\bar{\varphi }}^{T}$), mean coancestry (${\bar{\theta }}^{T}$), or bias coefficient ($s^{T}=\frac{{\bar{\theta }}^{T}}{F_{\text{ST}}}$ for IAFs, replaced by $\frac{{\bar{\varphi }}^{T}}{F_{\text{ST}}}$ for genotypes) are usually unknown. (A) Estimation from individual-specific allele frequencies (IAFs): ${\hat{F}}_{\text{ST}}^{\text{std}}$ is the standard coancestry plug-in estimator in Eq (26); ${\hat{F}}_{\text{ST}}^{\prime }$ “Adj. ${\bar{\theta }}^{T}$” is in Eq (27); ${\hat{F}}_{\text{ST}}^{\prime \prime }$ “Adj. *s*” is in Eq (31). (B) For genotypes, ${\hat{F}}_{\text{ST}}^{\text{std}}$ is given in Eq (25), and the adjusted estimators use ${\bar{\varphi }}^{T}$ rather than ${\bar{\theta }}^{T}$. Lines: true *F*_ST_ (red line), limits of biased estimators ${\hat{F}}_{\text{ST}}^{\text{std}}$ (green lines, which differ slightly per panel). Estimates (blue) include 95% prediction intervals (too narrow to see) from 39 independently-simulated genotype matrices for our admixture model (Methods, section **Prediction intervals**).

Here we verify additional calculations for the bias of the standard kinship-based estimator ${\hat{F}}_{\text{ST}}^{\text{std}}$ and the unbiased adjusted “oracle” *F*_ST_ estimators that require the true mean kinship ${\bar{\varphi }}^{T}$ or the bias coefficient *s*^*T*^ to be known. Note that ${\hat{F}}_{\text{ST}}^{\text{new}}$ in Eq (36) is related but not identical to these oracle estimators. We tested both IAF (Fig 7A) and genotype (Fig 7B) versions of these estimators. The unadjusted ${\hat{F}}_{\text{ST}}^{\text{std}}$ in Eq (26) is severely biased (blue in Fig 7) by construction, and matches the calculated limit for IAFs and genotypes (green lines in Fig 7, which are close because ${\bar{\varphi }}^{T}\approx {\bar{\theta }}^{T}$). In contrast, the two consistent adjusted estimators ${\hat{F}}_{\text{ST}}^{\prime }$ and ${\hat{F}}_{\text{ST}}^{\prime \prime }$ in Eqs (27) and (31) estimate *F*_ST_ quite well (blue predictions overlap the true *F*_ST_ red line in Fig 7). However, ${\hat{F}}_{\text{ST}}^{\prime }$ and ${\hat{F}}_{\text{ST}}^{\prime \prime }$ are oracle methods, since they require parameters (${\bar{\varphi }}^{T}$, ${\bar{\theta }}^{T}$, *s*^*T*^) that are not known in practice.

Prediction intervals were computed from estimates over 39 independently-simulated IAF and genotype matrices (Methods, section **Prediction intervals**). Estimator limits are always contained in these intervals because the number of independent loci (*m* = 300, 000) is sufficiently large. Estimates that use genotypes have wider intervals than estimates from IAFs; however, IAFs are not known in practice, and use of estimated IAFs might increase noise. Genetic linkage, not present in our simulation, will also increase noise in real data.

## Discussion

We studied analytically the most commonly-used estimators of *F*_ST_ and kinship, which can be derived using the method of moments. We determined the estimation limits of convergence of these approaches under two models of arbitrary population structure (Fig 1). We found that no existing approaches estimate the generalized *F*_ST_ (an IBD probability) accurately (but note that some of these approaches intended to estimate a linearly-transformed *F*_ST_ quantity and not the IBD probability). We also showed that the standard kinship estimator is biased on structured populations (particularly when the average kinship is comparable to the kinship coefficients of interest), and this bias varies for each pair of individuals. These results led us to a new kinship estimator, which is consistent if the minimum kinship is estimated consistently (Fig 1). We presented an implementation of this approach, which is practically unbiased in our simulations. Our kinship and *F*_ST_ estimates in human data are consistent with the African Origins model while suggesting that human differentiation is considerably greater than previously estimated [59].

Estimation of *F*_ST_ in the correct scale is crucial for its interpretation as an IBD probability, for obtaining comparable estimates in different datasets and across species, as well as for DNA forensics [3, 7, 19, 20, 78–80]. Our framework results in a new unbiased genome-wide *F*_ST_ estimator. However, our findings may not have direct implications for single-locus *F*_ST_ estimate approaches where only the relative ranking matters, such as for the identification of loci under selection [8, 10, 81–86], assuming that the bias of the genome-wide estimator carries over uniformly to all single-locus estimates. Our convergence calculations in section **Assessing the accuracy of genome-wide ratio estimators** require large numbers of loci, so they do not apply to single-locus estimates. Moreover, various methods for single-locus *F*_ST_ estimation for multiple alleles suffer from a strong dependence to the maximum allele frequency and heterozygosity [83–85, 87–90] that suggests that a more complicated bias is present in these single-locus *F*_ST_ estimators.

We have shown that the misapplication of existing *F*_ST_ estimators for independent subpopulations may lead to downwardly-biased estimates that can approach zero even when the true generalized *F*_ST_ is large. Weir-Cockerham [17], Weir-Hill [4], HudsonK (which generalizes the Hudson pairwise *F*_ST_ estimator [23] to *K* independent populations; also equals the Weir-Goudet approach for subpopulations [21]; S1 Text), and BayeScan [10]*F*_ST_ estimates in our admixture simulation are all smaller than the *F*_ST_ target by nearly a factor of two (Fig 4B), and differ from our new *F*_ST_ estimates in humans by nearly a factor of three [59]. To be accurate, existing *F*_ST_ estimators require independent subpopulations, so the observed biases arise from their misapplication to subpopulations that are neither independent not homogeneous. Nevertheless, natural populations—particularly humans—often do not adhere to the independent subpopulations model [59, 91–95].

The standard kinship coefficient estimator we investigated is often used to control for population structure in GWAS and to estimate genome-wide heritability [16, 27, 30–35]. While this estimator was known to be biased [16, 35], no closed-form limit had been calculated until very recently [21, 62]. These kinship estimates are biased downwards on average, but bias also varies for each pair of individuals (Figs 1 and 5). Thus, the use of these distorted kinship estimates may be problematic in GWAS or for estimating heritability, but the extent of the problem remains to be determined.

We developed a theoretical framework for assessing genome-wide ratio estimators of *F*_ST_ and kinship. We proved that common ratio-of-means estimators converge almost surely to the ratio of expectations for infinite independent loci (S1 Text). Our result justifies approximating the expectation of a ratio-of-means estimator with the ratio of expectations [4, 17, 23]. However, mean-of-ratios estimators may not converge to the ratio of expectations for infinite loci. Mean-of-ratios estimators are potentially asymptotically unbiased for infinite individuals, but it is unclear which estimators have this behavior. We found that the ratio-of-means kinship estimator had much smaller errors from the ratio of expectations than the more common mean-of-ratios estimator, whose convergence value is unknown. Therefore, we recommend ratio-of-means estimators, whose asymptotic behavior is well understood.

Our new framework enables accurate *F*_ST_ estimation in more complex datasets than before, but challenges remain. One challenge is the estimation of local inbreeding coefficients, which are required for estimating the generalized *F*_ST_ when not all individuals are locally outbred. To this end, we suggest employing existing approaches that infer inbreeding from large runs of homozygosity or related strategies [66–68], particularly when such self-IBD blocks are much larger than observed between individuals in the same subpopulation. A streamlined approach for jointly estimating total and local inbreeding is desirable, but will require an appropriate evaluation featuring realistic simulation of local inbreeding in a complex population structure. Another challenge is the estimation of the minimum kinship value without the use of subpopulation labels, so that accurate *F*_ST_ estimates can be obtained with even less user supervision. A more general unsupervised method could better ensure accuracy under extreme cases, such as when there are few unrelated individual pairs. These challenges can be overcome with the estimators we have presented, although supervision is needed to ensure that local inbreeding and the minimum kinship are estimated correctly.

We have demonstrated the need for new models and methods to study complex population structures, and have proposed a new approach for kinship and *F*_ST_ estimation that provides nearly unbiased estimates in this setting. Extending our implementation to deliver consistent accuracy in arbitrary population structures will require further innovation, and the results provided here may be useful in leading to more robust estimators in the future.

## Methods

### Previous *F*_ST_ estimators for the independent subpopulations model

Here we summarize the previous Weir-Cockerham, Weir-Hill, and Hudson *F*_ST_ estimators for independent subpopulations and derive the generalized HudsonK estimator for more than two subpopulations (which also equals the recent Weir-Goudet *F*_ST_ estimator for subpopulations under biallelic loci; S1 Text). We show that each of these estimators reduces, under infinite subpopulation sizes, to ${\hat{F}}_{\text{ST}}^{\text{indep}}$ in Eqs (11) to (13) that was studied in the results. In this section, let *i* index the *m* loci, *j* index the *n* subpopulations, *n*_*j*_ be the number of individuals sampled from subpopulation *j*, and ${\hat{p}}_{ij}$ be the sample reference allele frequency at locus *i* in subpopulation *j*.

#### The Weir-Cockerham *F*_ST_ estimator

The Weir-Cockerham (WC) *F*_ST_ estimator [17] estimates the coancestry parameter *θ*^*T*^ shared by each of the *n* independent subpopulation in consideration. Let ${\hat{h}}_{ij}$ denote the fraction of heterozygotes in subpopulation *j* for locus *i*. The ratio-of-means WC *F*_ST_ estimator and its limit for independent subpopulations ($\theta_{jk}^{T}=0$ for *j* ≠ *k*) with equal differentiation ($\theta_{jj}^{T}=\theta^{T}$) is

$$\begin{array}{cc} \bar{n} & =\frac{1}{n}\sum_{j=1}^{n}n_{j},\;C^{2}=\frac{1}{{\bar{n}}^{2}\left(n-1\right)}\sum_{j=1}^{n}{\left(n_{j}-\bar{n}\right)}^{2},\\ {\hat{p}}_{i}^{T} & =\frac{1}{n}\sum_{j=1}^{n}\frac{n_{j}}{\bar{n}}{\hat{p}}_{ij},\;{\bar{h}}_{i}=\frac{1}{n}\sum_{j=1}^{n}\frac{n_{j}}{\bar{n}}{\hat{h}}_{ij},\;{\hat{\sigma }}_{i}^{2}=\frac{1}{n-1}\sum_{j=1}^{n}\frac{n_{j}}{\bar{n}}{\left({\hat{p}}_{ij}-{\hat{p}}_{i}^{T}\right)}^{2},\\ {\hat{F}}_{\text{ST}}^{\text{WC}} & =\frac{\sum_{i=1}^{m}{\hat{\sigma }}_{i}^{2}-\frac{1}{\bar{n}-1}({\hat{p}}_{i}^{T}(1-{\hat{p}}_{i}^{T})-\frac{n-1}{n}{\hat{\sigma }}_{i}^{2}-\frac{1}{4}{\bar{h}}_{i})}{\sum_{i=1}^{m}{\hat{p}}_{i}^{T}(1-{\hat{p}}_{i}^{T})(1-\frac{\bar{n}C^{2}}{n(\bar{n}-1)})+\frac{1}{n}{\hat{\sigma }}_{i}^{2}(1+\frac{\left(n-1\right)\bar{n}C^{2}}{n(\bar{n}-1)})+\frac{{\bar{h}}_{i}C^{2}}{4n(\bar{n}-1)}}\\ & \overset{\text{a.s.}}{\underset{m\to \infty }{\to }}F_{\text{ST}}=\theta^{T}. \end{array}$$

Note that ${\hat{p}}_{i}^{T}$ above weighs every individual equally by weighing subpopulation *j* proportional to its sample size *n*_*j*_, so it equals the estimator in Eq (17) with uniform weights.

Now we simplify this estimator as the sample size of every subpopulation becomes infinite. First set the sample size of every subpopulation *n*_*j*_ equal to their mean $\bar{n}$, which implies *C*^2^ = 0 and

$$\begin{array}{cc} {\hat{p}}_{i}^{T} & =\frac{1}{n}\sum_{j=1}^{n}{\hat{p}}_{ij},\;{\bar{h}}_{i}=\frac{1}{n}\sum_{j=1}^{n}{\hat{h}}_{ij},\;{\hat{\sigma }}_{i}^{2}=\frac{1}{n-1}\sum_{j=1}^{n}{\left({\hat{p}}_{ij}-{\hat{p}}_{i}^{T}\right)}^{2},\\ {\hat{F}}_{\text{ST}}^{\text{WC}} & =\frac{\sum_{i=1}^{m}{\hat{\sigma }}_{i}^{2}-\frac{1}{\bar{n}-1}({\hat{p}}_{i}^{T}(1-{\hat{p}}_{i}^{T})-\frac{n-1}{n}{\hat{\sigma }}_{i}^{2}-\frac{1}{4}{\bar{h}}_{i})}{\sum_{i=1}^{m}{\hat{p}}_{i}^{T}(1-{\hat{p}}_{i}^{T})+\frac{1}{n}{\hat{\sigma }}_{i}^{2}}. \end{array}$$

Now we take the limit as the sample size $\bar{n}\to \infty$, which results in sample allele frequencies converging to the true subpopulation allele frequencies ${\hat{p}}_{ij}\to \pi_{ij}$ for every subpopulation *j* and locus *i*, and

$${\hat{p}}_{i}^{T}=\frac{1}{n}\sum_{j=1}^{n}\pi_{ij},\;{\hat{\sigma }}_{i}^{2}=\frac{1}{n-1}\sum_{j=1}^{n}{\left(\pi_{ij}-{\hat{p}}_{i}^{T}\right)}^{2},\;{\hat{F}}_{\text{ST}}^{\text{WC}}=\frac{\sum_{i=1}^{m}{\hat{\sigma }}_{i}^{2}}{\sum_{i=1}^{m}{\hat{p}}_{i}^{T}(1-{\hat{p}}_{i}^{T})+\frac{1}{n}{\hat{\sigma }}_{i}^{2}},$$

which matches the ${\hat{F}}_{\text{ST}}^{\text{indep}}$ in Eqs (11) to (13) as desired. Note the number of subpopulations *n* remains finite, and the sample heterozygosity ${\bar{h}}_{i}$ is not needed in the limit.

#### The Weir-Hill *F*_ST_ estimator

Weir and Hill developed new estimators for subpopulation-specific *F*_ST_ values and considered the effects of non-independent subpopulations [4]. However, these estimators target linearly-transformed *F*_ST_ values, and recover the *F*_ST_ defined in Eq (9) only when subpopulations are independent [4], so we group them here with other estimators that strictly assume independent subpopulations. For simplicity, here we only consider the global *F*_ST_ estimator; the estimators of the coancestry matrix of the subpopulations was found to have the same overall linear transformation [4]. In the limit of infinite subpopulation sizes, this estimator also converges to the asymptotic *F*_ST_ estimator for independent subpopulations (${\hat{F}}_{\text{ST}}^{\text{indep}}$) discussed in the main text.

The Weir-Hill (WH) *F*_ST_ estimator, simplified here for biallelic loci but extended to average over loci, and its limit, are given by

$$\begin{array}{cc} {\hat{p}}_{i}^{T} & =\sum_{j=1}^{n}w_{j}{\hat{p}}_{ij},\;\;w_{j}=\frac{n_{j}}{\sum_{j=1}^{n}n_{j}},\\ {\hat{F}}_{\text{ST}}^{\text{WH}} & =1-\frac{\left(\sum_{j=1}^{n}n_{j}\left(1-w_{j}\right))(\sum_{i=1}^{m}\sum_{j=1}^{n}w_{j}\frac{2n_{j}}{2n_{j}-1}{\hat{p}}_{ij}(1-{\hat{p}}_{ij})\right)}{\sum_{i=1}^{m}\sum_{j=1}^{n}n_{j}{\left({\hat{p}}_{ij}-{\hat{p}}_{i}^{T}\right)}^{2}+n_{j}\left(1-w_{j}\right){\hat{p}}_{ij}(1-{\hat{p}}_{ij})}\overset{\text{a.s.}}{\underset{m\to \infty }{\to }}\frac{F_{\text{ST}}-{\tilde{\theta }}^{T}}{1-{\tilde{\theta }}^{T}}, \end{array}$$

where the target *F*_ST_ and ${\tilde{\theta }}^{T}$ both weigh individuals (rather than subpopulations) equally [4]:

$$\begin{array}{c} F_{\text{ST}}=\sum_{j=1}^{n}w_{j}\theta_{jj}^{T},\;\;{\tilde{\theta }}^{T}=\frac{2}{1-\sum_{j=1}^{n}w_{j}^{2}}\sum_{j=2}^{n}\sum_{k=1}^{j-1}w_{j}w_{k}\theta_{jk}^{T}. \end{array}$$

For equal sample sizes *n*_*j*_ = *n*_*S*_∀*j*, we have $w_{j}=\frac{1}{n}$, $n_{jc}=n_{S}(1-\frac{1}{n})$, and the estimator becomes

$$\begin{array}{cc} {\hat{p}}_{i}^{T} & =\frac{1}{n}\sum_{j=1}^{n}{\hat{p}}_{ij},\;{\hat{\sigma }}_{i}^{2}=\frac{1}{n-1}\sum_{j=1}^{n}{\left({\hat{p}}_{ij}-{\hat{p}}_{i}^{T}\right)}^{2},\\ {\hat{F}}_{\text{ST}}^{\text{WH}} & =\frac{\sum_{i=1}^{m}{\hat{\sigma }}_{i}^{2}(\frac{2n_{S}-\frac{1}{n}}{2n_{s}-1})-p_{i}^{T}(1-p_{i}^{T})\left(\frac{1}{2n_{s}-1}\right)}{\sum_{i=1}^{m}p_{i}^{T}(1-p_{i}^{T})+\frac{1}{n}{\hat{\sigma }}_{i}^{2}}. \end{array}$$

Therefore, as sample sizes per subpopulation go to infinity (*n*_*S*_ → ∞, which results in ${\hat{p}}_{ij}\to \pi_{ij}$ for every (*i*, *j*)), we again recover the desired limiting *F*_ST_ estimator for independent subpopulations (${\hat{F}}_{\text{ST}}^{\text{indep}}$ in Eqs (11) to (13)).

#### The Hudson *F*_ST_ estimator

The Hudson pairwise *F*_ST_ estimator [23] measures the differentiation of two subpopulations (*j*, *k*). The estimator and its limit for two independent subpopulations ($\theta_{jk}^{T}=0$) is

$$\begin{array}{c} {\hat{F}}_{\text{ST}}^{\text{Hudson}}=\frac{\sum_{i=1}^{m}{\left({\hat{p}}_{ij}-{\hat{p}}_{ik}\right)}^{2}-\frac{{\hat{p}}_{ij}(1-{\hat{p}}_{ij})}{2n_{j}-1}-\frac{{\hat{p}}_{ik}(1-{\hat{p}}_{ik})}{2n_{k}-1}}{\sum_{i=1}^{m}{\hat{p}}_{ij}(1-{\hat{p}}_{ik})+{\hat{p}}_{ik}(1-{\hat{p}}_{ij})}\overset{\text{a.s.}}{\underset{m\to \infty }{\to }}F_{\text{ST}}=\frac{\theta_{jj}^{T}+\theta_{kk}^{T}}{2}. \end{array}$$ (40)

#### Generalized HudsonK *F*_ST_ estimator

Here we derive the “HudsonK” estimator (first made available in [58]), which generalizes the Hudson pairwise *F*_ST_ estimator in Eq (40) to *n* independent subpopulations. This estimator also equals the recent Weir-Goudet *F*_ST_ estimator for subpopulations [21] (for biallelic loci; S1 Text). Note that for independent subpopulations, the *F*_ST_ of all the subpopulations equals the mean pairwise *F*_ST_ of every pair of subpopulations:

$$\begin{array}{c} \frac{1}{n^{2}}\sum_{j=1}^{n}\sum_{k=1}^{n}(\frac{\theta_{jj}^{T}+\theta_{kk}^{T}}{2})=\frac{1}{n}\sum_{j=1}^{n}\theta_{jj}^{T}=F_{\text{ST}}. \end{array}$$

For that reason, averaging numerators and denominators of the pairwise estimator in Eq (40) before computing the ratio, we obtain the generalized estimator and a limit under independent subpopulations of

$$\begin{array}{cc} {\hat{p}}_{i}^{T} & =\frac{1}{n}\sum_{j=1}^{n}{\hat{p}}_{ij},\;{\hat{\sigma }}_{i}^{2}=\frac{1}{n-1}\sum_{j=1}^{n}{\left({\hat{p}}_{ij}-{\hat{p}}_{i}^{T}\right)}^{2},\\ {\hat{F}}_{\text{ST}}^{\text{HudsonK}} & =\frac{\sum_{i=1}^{m}{\hat{\sigma }}_{i}^{2}-\frac{1}{n}\sum_{j=1}^{n}\frac{{\hat{p}}_{ij}(1-{\hat{p}}_{ij})}{2n_{j}-1}}{\sum_{i=1}^{m}{\hat{p}}_{i}^{T}\left(1-{\hat{p}}_{i}^{T}\right)+\frac{1}{n}{\hat{\sigma }}_{i}^{2}}\overset{\text{a.s.}}{\underset{m\to \infty }{\to }}F_{\text{ST}}=\frac{1}{n}\sum_{j=1}^{n}\theta_{jj}^{T}. \end{array}$$

Note that unlike the WC and Weir-Hill estimators, ${\hat{p}}_{i}^{T}$ above weighs every subpopulation equally, so every individual is weighed inversely proportional to the sample sizes *n*_*j*_ of their subpopulation *j*.

Like WC and Weir-Hill, ${\hat{F}}_{\text{ST}}^{\text{HudsonK}}$ simplifies to ${\hat{F}}_{\text{ST}}^{\text{indep}}$ in Eqs (11) to (13) in the limit of infinite sample sizes *n*_*j*_ → ∞, where ${\hat{p}}_{ij}\to \pi_{ij}$ for every (*i*, *j*).

### Simulations

#### Construction of subpopulation allele frequencies

We simulate *K* = 10 subpopulations *S*_*u*_ and *m* = 300, 000 independent loci. Every locus *i* draws $p_{i}^{T}\sim \text{Uniform}(0.01,0.5).$ We set $f_{S_{u}}^{T}=\frac{u}{K}\tau ,$ where *τ* ≤ 1 tunes *F*_ST_. For the independent subpopulations model, $F_{\text{ST}}=\frac{1}{K}\sum_{u=1}^{K}f_{S_{u}}^{T}=\frac{\tau (K+1)}{2K},$ so $\tau =\frac{2KF_{\text{ST}}}{K+1}$ gives the desired *F*_ST_ (*τ* ≈ 0.18 for *F*_ST_ = 0.1). For the admixture model, *τ* is found numerically (*τ* ≈ 0.90 for *F*_ST_ = 0.1; see last subsection). Lastly, $p_{i}^{S_{u}}$ values are drawn from the Balding-Nichols distribution,

$$p_{i}^{S_{u}}|T∼\text{Beta}(p_{i}^{T}(\frac{1}{f_{S_{u}}^{T}}-1),\left(1-p_{i}^{T}\right)(\frac{1}{f_{S_{u}}^{T}}-1)),$$

which results in subpopulation allele frequencies that obey the coancestry model of Eq (6), with $E[p_{i}^{S_{u}}|T]=p_{i}^{T}$ and $\mathrm{Var}(p_{i}^{S_{u}}|T)=f_{S_{u}}^{T}p_{i}^{T}(1-p_{i}^{T})$ [3], as desired.

#### Random subpopulation sizes

We randomly generate sample sizes **r** = (*r*_*u*_) for *K* subpopulations and $\sum_{u=1}^{K}r_{u}=n=1000$ individuals, as follows. First, draw **x** ∼ Dirichlet (1, …, 1) of length *K* and **r** = round(*n* **x**). While $\min_{u}r_{u}<\frac{n}{3K}$, draw a new **r**, to prevent small subpopulations (they do not occur in real data). Due to rounding, $\sum_{u=1}^{K}r_{u}$ may not equal *n* as desired. Thus, while $\delta =n-\sum_{u=1}^{K}r_{u}\neq 0$, a random *u* is updated to *r*_*u*_ ← *r*_*u*_ + sgn(*δ*), which brings *δ* closer to zero at every iteration. Weights for individuals *j* in *S*_*u*_ are $w_{j}=\frac{1}{Kr_{u}}$ so the generalized *F*_ST_ matches $F_{\text{ST}}=\frac{1}{K}\sum_{u=1}^{K}f_{S_{u}}^{T}$ from the independent subpopulations model (see section **The generalized *F*_ST_ for arbitrary population structures**), which HudsonK estimates.

#### Admixture proportions from 1D geography

We construct *q*_*ju*_ from random-walk migrations along a one-dimensional geography. Let *x*_*u*_ be the coordinate of intermediate subpopulation *u* and *y*_*j*_ the coordinate of a modern individual *j*. We assume *q*_*ju*_ is proportional to *f*(|*x*_*u*_ − *y*_*j*_|), or

$$\begin{array}{c} q_{ju}=\frac{f(|x_{u}-y_{j}|)}{\sum_{v=1}^{K}f(|x_{v}-y_{j}\left|\right)}. \end{array}$$

where *f* is the Normal density function with *μ* = 0 and tunable *σ*. The Normal density models random walks, where *σ* sets the spread of the populations (Fig 5). Our simulation uses *x*_*u*_ = *u* and $y_{j}=\frac{1}{2}+\frac{j-1}{n-1}K$, so the intermediate subpopulations span between 1 and *K* and individuals span between $\frac{1}{2}$ and $K+\frac{1}{2}$. For the *F*_ST_ estimators that require subpopulations, individual *j* is assigned to the nearest subpopulation *S*_*u*_ (the *u* that minimizes |*x*_*u*_ − *y*_*j*_|; Fig 3D); these subpopulations have equal sample size, so $w_{j}=\frac{1}{n}$ is appropriate.

#### Choosing *σ* and *τ*

Here we find values for *σ* (controls *q*_*jk*_) and *τ* (scales $f_{S_{u}}^{T}$) that give $s^{T}=\frac{1}{2}$ and *F*_ST_ = 0.1 in the admixture model. In our simulation, $w_{j}=\frac{1}{n}$ and $f_{S_{u}}^{T}=\frac{u}{K}\tau$, so applying those parameters to Eq (39) gives $\theta_{jk}^{T}=\frac{\tau }{K}\sum_{u=1}^{K}uq_{ju}q_{ku}$ and $F_{\text{ST}}=\frac{\tau }{nK}\sum_{j=1}^{n}\sum_{u=1}^{K}uq_{ju}^{2}$. Therefore,

$$\begin{array}{c} s^{T}=\frac{{\bar{\theta }}^{T}}{F_{\text{ST}}}=\frac{1}{n}\frac{\sum_{u=1}^{K}u{\left(\sum_{j=1}^{n}q_{ju}\left(\sigma \right)\right)}^{2}}{\sum_{u=1}^{K}u(\sum_{j=1}^{n}q_{ju}^{2}\left(\sigma \right))} \end{array}$$

depends only on *σ*. A numerical root finder finds that *σ* ≈ 1.78 gives $s^{T}=\frac{1}{2}$. For fixed *q*_*ju*_,

$$\begin{array}{c} \tau =\frac{F_{\text{ST}}}{\frac{1}{K}\sum_{u=1}^{K}u(\frac{1}{n}\sum_{j=1}^{n}q_{ju}^{2})}. \end{array}$$

*F*_ST_ = 0.1 is achieved with *τ* ≈ 0.901.

### Prediction intervals

Prediction intervals with *α* = 95% correspond to the range of *n* = 39 independent *F*_ST_ estimates. In the general case, *n* independent statistics are given in order *X*_(1)_ < … < *X*_(*n*)_. Then *I* = [*X*_(*j*)_, *X*_(*n*+1−*j*)_] is a prediction interval with confidence $\alpha =\frac{n+1-2j}{n+1}$ [96]. In our case, *j* = 1 and *n* = 39 gives *α* = 0.95, as desired. Each estimate was constructed from simulated data with the same dimensions and structure as before (fixed $f_{S_{u}}^{T}$ and *q*_*ju*_; fixed sample sizes for the independent subpopulations model), but with $p_{i}^{T},p_{i}^{S_{u}},\pi_{ij},x_{ij}$ drawn separately for each estimate.

### BayeScan and Weir-Goudet implementations

Weir-Goudet (WG) kinship estimates [20–22] were calculated using the function snpgdsIndivBeta in the R package SNPRelate 1.20.1 available on Bioconductor and GitHub. We found identical estimates using the function beta.dosage in the R package hierfstat 0.4.30 available on GitHub. WG (individuals) *F*_ST_ estimates were computed from the kinship estimates as described in section **Comparison to the Weir-Goudet kinship estimator for individuals**.

BayeScan 2.1 was downloaded from http://cmpg.unibe.ch/software/BayeScan/. To estimate *F*_ST_, first the per-subpopulation *F*_ST_ values were estimated across loci assuming no selection, then the global *F*_ST_ was given by the mean *F*_ST_ across subpopulations.

### Software

An R package called popkin, which implements the kinship and *F*_ST_ estimation methods proposed here, is available on the Comprehensive R Archive Network (CRAN) at https://cran.r-project.org/package=popkin and on GitHub at https://github.com/StoreyLab/popkin.

An R package called bnpsd, which implements the BN-PSD admixture simulation, is available on CRAN at https://cran.r-project.org/package=bnpsd and on GitHub at https://github.com/StoreyLab/bnpsd.

An R package called popkinsuppl, which implements memory-efficient algorithms for the Weir-Cockerham, Weir-Hill, and HudsonK *F*_ST_ estimators, and the standard kinship estimator, is available on GitHub at https://github.com/OchoaLab/popkinsuppl.

Public code reproducing these analyses are available at https://github.com/StoreyLab/human-differentiation-manuscript.

## Supporting information

S1 Text Supplementary information. Includes mathematical proofs and other calculations, including proof of convergence of ratio-of-means estimators, proof that the Weir-Goudet *F*_ST_ estimator for subpopulations equals HudsonK, derivation of existing method-of-moment estimators, proof that *F*_ST_ and kinship estimator limits are constants with respect to the ancestral population *T*, mean coancestry bounds, moments of estimator building blocks, the derivation of our new kinship estimator, and proof that our estimator from our original 2016 manuscript is algebraically equivalent to the one presented here.
(PDF)
