# Supplementary material for: Estimating FST and kinship for arbitrary population structures
Source: PLoS Genet. 2021 Jan 19;17(1):e1009241. doi: 10.1371/journal.pgen.1009241 (PMC7846127; doi:10.1371/journal.pgen.1009241)
Supplement: S1 Text — Includes mathematical proofs and other calculations, including proof of convergence of ratio-of-means estimators, proof that the Weir-Goudet FST estimator for subpopulations equals HudsonK, derivation of existing method-of-moment estimators, proof that FST and kinship estimator limits are constants with respect to the ancestral population T, mean coancestry bounds, moments of estimator building blocks, the derivation of our new kinship estimator, and proof that our estimator from our original 2016 manuscript is algebraically equivalent to the one presented here. (PDF) [file pgen.1009241.s001.pdf]

Supplementary Information:
Estimating F_{ST} and kinship for arbitrary population structures

Alejandro Ochoa[1,2] and John D. Storey[3,*]

[1]Duke Center for Statistical Genetics and Genomics, and [2]Department of Biostatistics and Bioinformatics, Duke University, Durham, NC 27705, USA

[3]Lewis-Sigler Institute for Integrative Genomics, Princeton University, Princeton, NJ 08544, USA

∗ Corresponding author: jstorey@princeton.edu

Contents

A Accuracy of ratio estimators **S2**

A.1 Almost sure convergence of ratio-of-means estimators with independent and uniformly-bounded terms . . . S2

A.2 Order of error of expectations . . . S2

B The Weir-Goudet F_{ST} estimator for subpopulations **S2**

C Derivation of existing method-of-moment estimators **S5**

C.1 F_{ST} estimator for independent subpopulations . . . S5

C.2 Standard kinship estimator . . . S6

D Proofs that F_{ST} and kinship estimator limits are constants with respect to the ancestral population T **S7**

D.1 Proof that the limit of $\hat{F}_{\text{ST}}^{\text{indep}}$ does not depend on T . . . S7

D.2 Proof that the limit of $\hat{\varphi}_{jk}^{T,\text{std}}$ does not depend on T . . . S7

E Mean coancestry bounds **S8**

F Moments of estimator building blocks **S8**

G Derivation of new kinship estimator **S9**

H Mathematical equivalence between the proposed kinship estimator as described here and our original 2016 proposal **S10**

A Accuracy of ratio estimators

A.1 Almost sure convergence of ratio-of-means estimators with independent and uniformly-bounded terms

Here we prove that $\frac{\hat{A}_m}{\hat{B}_m} \xrightarrow[m\to\infty]{\text{a.s.}} \frac{A}{B}$, where $\hat{A}_m = \frac{1}{m}\sum_{i=1}^{m} a_i$ and $\hat{B}_m = \frac{1}{m}\sum_{i=1}^{m} b_i$ give the ratio-of-means estimator described in the main text. It suffices to prove $\hat{A}_m \xrightarrow[m\to\infty]{\text{a.s.}} Ac$ and $\hat{B}_m \xrightarrow[m\to\infty]{\text{a.s.}} Bc \neq 0$, from which the result follows using the continuous mapping theorem [1, 2]. The proof for $\hat{A}_m$ follows, which applies analogously to $\hat{B}_m$. Our a_i are independent but not identically distributed, since they depend on p_i^T that varies per locus, so the standard law of large numbers does not apply to $\hat{A}_m$. We show almost sure convergence using Kolmogorov's criterion for the Strong Law of Large Numbers [3], which is satisfied for bounded $\operatorname{Var}(a_i)$. Since $|a_i| \leq C < \infty$ for all i and some C (see main text), then $\operatorname{E}[a_i^2] \leq C^2$, so $\operatorname{Var}(a_i) \leq C^2$. Therefore, $\hat{A}_m \xrightarrow[m\to\infty]{\text{a.s.}} \lim_{m\to\infty} \operatorname{E}\left[\hat{A}_m\right] = Ac$, as desired.

A.2 Order of error of expectations

The error of the ratio of expectations from the expectation of the ratio is given by

$$\epsilon_m = \operatorname{E}\left[\frac{\hat{A}_m}{\hat{B}_m}\right] - \frac{\operatorname{E}[\hat{A}_m]}{\operatorname{E}[\hat{B}_m]} = -\frac{\operatorname{Cov}\left(\frac{\hat{A}_m}{\hat{B}_m}, \hat{B}_m\right)}{\operatorname{E}\left[\hat{B}_m\right]} = -\frac{1}{m^2 Bc}\sum_{i=1}^{m}\sum_{j=1}^{m}\operatorname{Cov}\left(\frac{a_i}{\hat{B}_m}, b_j\right),$$

which follows from $\operatorname{Cov}(X,Y) = \operatorname{E}[XY] - \operatorname{E}[X]\operatorname{E}[Y]$ and expanding the covariance [4]. Previous work on ratio estimators [4, 5] assumes IID a_i and b_i, which does not hold for SNP loci. Assuming independent loci ($\operatorname{Cov}(a_i, b_j) = 0$ for $i \neq j$) and large m so $\hat{B}_m \approx Bc$ is practically independent of any given a_i and b_j, then

$$\epsilon_m \approx -\frac{1}{mB^2c^2}\left[\frac{1}{m}\sum_{i=1}^{m}\operatorname{Cov}(a_i, b_i)\right].$$

Since a_i, b_i are bounded, $|\operatorname{Cov}(a_i, b_i)| \leq C^2$ for the same C of the previous section, so

$$|\epsilon_m| \leq \frac{C^2}{mB^2c^2},$$

for some large enough m and C. Hence $\epsilon_m = O\left(\frac{1}{m}\right)$ as is for standard ratio estimators [5].

B The Weir-Goudet F_{ST} estimator for subpopulations

Here we show that the relative F_{ST} estimator presented in [6] (denoted by $\hat{\beta}_{\text{WT}}$ in that work) for biallelic loci equals the HudsonK F_{ST} estimator in the main text. This estimator—the special case for biallelic loci only—is implemented in the function `snpgdsFst` with `method` option set to `W&H02`

in the R package `SNPRelate` by the authors of [6]; despite this method name, this estimator does not equal the Weir-Hill estimator in [7], but rather the estimator described below (verified both numerically and by looking at the source code). Note that Weir-Cockerham (`W&C84`) is the only other F_{ST} estimator implemented in that package as of this writing (other F_{ST} estimators proposed in [6] are not implemented). The earliest presentation of this exact estimator that we are aware of is [8], which came out the same year that we first described HudsonK [9].

The Weir-Goudet relative F_{ST} estimator for subpopulations is given by

$$\begin{aligned}
\hat{F}_{\text{ST}}^{\text{WGsubpops}} &= \frac{\widetilde{M}^W - \widetilde{M}^B}{1 - \widetilde{M}^B}, \quad \text{where} \\
\widetilde{M}^W &= \frac{1}{m} \sum_{i=1}^m \frac{1}{n} \sum_{j=1}^n \frac{2n_j}{2n_j - 1} \left(\sum_u \hat{p}_{iju}^2 \right) - \frac{1}{2n_j - 1}, \\
\widetilde{M}^B &= \frac{1}{m} \sum_{i=1}^m \frac{1}{n(n-1)} \sum_{j=1}^n \sum_{k=1, k \neq j}^n \sum_u \hat{p}_{iju} \hat{p}_{iku},
\end{aligned} \tag{S1}$$

and $\hat{p}_{iju}$ is the sample allele frequency at locus i for subpopulation j and allele u. As in the existing F_{ST} methods section, here n is the number of subpopulations and n_j is the number of individuals in subpopulation j.

In order to establish the connection between this and other F_{ST} estimators, we first generalize some of our earlier notation to be for more than two alleles. We can calculate the allele frequency variance term per allele u, or

$$\hat{\sigma}_{iu}^2 = \frac{1}{n-1} \sum_{j=1}^n \left(\hat{p}_{iju} - \hat{p}_{iu}^T \right)^2,$$

where $\hat{p}_{iu}^T = \frac{1}{n} \sum_{j=1}^n \hat{p}_{iju}$ is the sample allele frequency estimate for each allele u weighing subpopulations equally. Expanding the square and rearranging, we find that the sum of square subpopulation sample allele frequencies is given by

$$\sum_{j=1}^n \hat{p}_{iju}^2 = (n-1)\hat{\sigma}_{iu}^2 + n \left(\hat{p}_{iu}^T \right)^2.$$

Going back to the WG estimator for subpopulations in Eq. (S1), its parts can be restated as

$$\begin{aligned}
\widetilde{M}^W &= \frac{1}{m} \sum_{i=1}^m \frac{1}{n} \sum_{j=1}^n \left(\sum_u \hat{p}_{iju}^2 \right) - \frac{1}{2n_j - 1} \left(1 - \sum_u \hat{p}_{iju}^2 \right) \\
&= \frac{1}{m} \sum_{i=1}^m \left(\sum_u \frac{1}{n} \sum_{j=1}^n \hat{p}_{iju}^2 \right) - \frac{1}{n} \sum_{j=1}^n \frac{1}{2n_j - 1} \left(1 - \sum_u \hat{p}_{iju}^2 \right) \\
&= \frac{1}{m} \sum_{i=1}^m \left(\sum_u \frac{n-1}{n} \hat{\sigma}_{iu}^2 + \left(\hat{p}_{iu}^T \right)^2 \right) - \frac{1}{n} \sum_{j=1}^n \frac{1}{2n_j - 1} \left(1 - \sum_u \hat{p}_{iju}^2 \right).
\end{aligned}$$

Similarly,

$$\begin{aligned}
\widetilde{M}^B &= \frac{1}{m} \sum_{i=1}^m \sum_u \frac{1}{n(n-1)} \sum_{j=1}^n \hat{p}_{iju} \sum_{k=1, k \neq j}^n \hat{p}_{iku} \\
&= \frac{1}{m} \sum_{i=1}^m \sum_u \frac{1}{n(n-1)} \sum_{j=1}^n \hat{p}_{iju} \left(n\hat{p}_{iu}^T - \hat{p}_{iju} \right) \\
&= \frac{1}{m} \sum_{i=1}^m \sum_u \frac{1}{n(n-1)} \left(\left(n\hat{p}_{iu}^T \right)^2 - \sum_{j=1}^n \left(\hat{p}_{iju} \right)^2 \right) \\
&= \frac{1}{m} \sum_{i=1}^m \sum_u \frac{1}{n(n-1)} \left(\left(n\hat{p}_{iu}^T \right)^2 - (n-1)\hat{\sigma}_{iu}^2 - n \left(\hat{p}_{iu}^T \right)^2 \right) \\
&= \frac{1}{m} \sum_{i=1}^m \sum_u \left(\hat{p}_{iu}^T \right)^2 - \frac{1}{n} \hat{\sigma}_{iu}^2.
\end{aligned}$$

Now, taking the special case of biallelic loci ($\hat{p}_{iu}^T = \hat{p}_i^T$ for the first allele u, and $\hat{p}_{iu}^T = 1 - \hat{p}_i^T$ for the second allele; note that $\hat{\sigma}_{iu}^2 = \hat{\sigma}_i^2$ for both alleles; also note repeated use of the identity $p^2 + (1-p)^2 = 1 - 2p(1-p)$), the expressions simplify to

$$\begin{aligned}
\widetilde{M}^W &= \frac{1}{m} \sum_{i=1}^m \left(2 \frac{n-1}{n} \hat{\sigma}_i^2 + \left(p_i^T \right)^2 + \left(1 - p_i^T \right)^2 \right) - \frac{1}{n} \sum_{j=1}^n \frac{1}{2n_j - 1} \left(1 - \hat{p}_{ij}^2 - (1 - \hat{p}_{ij})^2 \right) \\
&= 1 + \frac{2}{m} \sum_{i=1}^m \frac{n-1}{n} \hat{\sigma}_i^2 - p_i^T \left(1 - p_i^T \right) - \frac{1}{n} \sum_{j=1}^n \frac{\hat{p}_{ij}(1 - \hat{p}_{ij})}{2n_j - 1}, \\
\widetilde{M}^B &= \frac{1}{m} \sum_{i=1}^m \left(\hat{p}_i^T \right)^2 + \left(1 - \hat{p}_i^T \right)^2 - \frac{2}{n} \hat{\sigma}_i^2 \\
&= 1 - \frac{2}{m} \sum_{i=1}^m \hat{p}_i^T \left(1 - \hat{p}_i^T \right) + \frac{1}{n} \hat{\sigma}_i^2.
\end{aligned}$$

Thus, the numerator and denominator of Eq. (S1), respectively, are also equal to

$$\begin{aligned}
\widetilde{M}^W - \widetilde{M}^B &= \frac{2}{m} \sum_{i=1}^m \hat{\sigma}_i^2 - \frac{1}{n} \sum_{j=1}^n \frac{\hat{p}_{ij}(1 - \hat{p}_{ij})}{2n_j - 1}, \\
1 - \widetilde{M}^B &= \frac{2}{m} \sum_{i=1}^m \hat{p}_i^T \left(1 - \hat{p}_i^T \right) + \frac{1}{n} \hat{\sigma}_i^2.
\end{aligned}$$

Thus, the above numerator and denominator are, respectively, twice the values of the HudsonK estimator in the main text, a common factor of two that cancels out. Therefore, in this special case where every locus is biallelic, the WG F_{ST} estimator for subpopulations equals the HudsonK estimator.

C Derivation of existing method-of-moment estimators

C.1 F_{ST} estimator for independent subpopulations

Assuming the coancestry model in Eq. (6) in the main text for independent subpopulations ($\theta^T_{jk} = 0$ for $j \neq k$), the first and second moments of the IAFs are:

$$\mathrm{E}[\pi_{ij}|T] = p^T_i, \tag{S2}$$

$$\mathrm{E}\left[\pi^2_{ij}|T\right] = \left(p^T_i\right)^2 + p^T_i\left(1 - p^T_i\right)\theta^T_{jj}, \tag{S3}$$

$$\mathrm{E}\left[\pi_{ij}\pi_{ik}|T\right] = \left(p^T_i\right)^2 \quad \text{if} \quad j \neq k. \tag{S4}$$

$F_{\mathrm{ST}} = \frac{1}{n}\sum_{j=1}^{n}\theta^T_{jj}$ appears by averaging Eq. (S3) over j:

$$\mathrm{E}\left[\frac{1}{n}\sum_{j=1}^{n}\pi^2_{ij}\middle|T\right] = \left(p^T_i\right)^2 + p^T_i\left(1 - p^T_i\right)F_{\mathrm{ST}}. \tag{S5}$$

Since Eq. (S2) has the same value for every j, and Eq. (S4) as well for every $j \neq k$, we average these to reduce estimation variance. The results are in terms of $\hat{p}^T_i = \frac{1}{n}\sum_{j=1}^{n}\pi_{ij}$:

$$\mathrm{E}\left[\hat{p}^T_i|T\right] = \mathrm{E}\left[\frac{1}{n}\sum_{j=1}^{n}\pi_{ij}\middle|T\right] = p^T_i, \tag{S6}$$

$$\mathrm{E}\left[\left(\hat{p}^T_i\right)^2\middle|T\right] = \mathrm{E}\left[\frac{1}{n^2}\sum_{j=1}^{n}\sum_{k=1}^{n}\pi_{ij}\pi_{ik}\middle|T\right] = \left(p^T_i\right)^2 + p^T_i\left(1 - p^T_i\right)\frac{1}{n}F_{\mathrm{ST}}. \tag{S7}$$

F_{ST} also appears in Eq. (S7) because $j = k$ terms are introduced in the double sum. Subtracting Eq. (S5) and Eq. (S7) in turn from Eq. (S6) results in:

$$\mathrm{E}\left[\hat{p}^T_i - \frac{1}{n}\sum_{j=1}^{n}\pi^2_{ij}\middle|T\right] = p^T_i\left(1 - p^T_i\right)\left(1 - F_{\mathrm{ST}}\right),$$

$$\mathrm{E}\left[\hat{p}^T_i\left(1 - \hat{p}^T_i\right)|T\right] = p^T_i\left(1 - p^T_i\right)\left(1 - \frac{1}{n}F_{\mathrm{ST}}\right).$$

To reduce variance further, we average across loci, giving

$$\mathrm{E}\left[\frac{1}{m}\sum_{i=1}^{m}\left(\hat{p}^T_i - \frac{1}{n}\sum_{j=1}^{n}\pi^2_{ij}\right)\middle|T\right] = \overline{p(1-p)}^T\left(1 - F_{\mathrm{ST}}\right),$$

$$\mathrm{E}\left[\frac{1}{m}\sum_{i=1}^{m}\hat{p}^T_i\left(1 - \hat{p}^T_i\right)\middle|T\right] = \overline{p(1-p)}^T\left(1 - \frac{1}{n}F_{\mathrm{ST}}\right),$$

where $\overline{p(1-p)}^T = \frac{1}{m}\sum_{i=1}^{m} p_i^T\left(1-p_i^T\right)$. Eliminating $\overline{p(1-p)}^T$ and solving for F_{ST} in this system of equations results in the following F_{ST} estimator:

$$\hat{F}_{\text{ST}}^{\text{indep}} = \frac{\sum_{i=1}^{m}\left(\frac{1}{n}\sum_{j=1}^{n}\pi_{ij}^2 - \left(\hat{p}_i^T\right)^2\right)}{\sum_{i=1}^{m}\left(\hat{p}_i^T\left(1-\hat{p}_i^T\right) + \frac{1}{n}\left(\frac{1}{n}\sum_{j=1}^{n}\pi_{ij}^2 - \hat{p}_i^T\right)\right)} \tag{S8}$$

This estimator is simplified noting that $\frac{1}{n}\sum_{j=1}^{n}\pi_{ij}^2$ appears in the IAF sample variance,

$$\hat{\sigma}_i^2 = \frac{1}{n-1}\sum_{j=1}^{n}\left(\pi_{ij} - \hat{p}_i^T\right)^2 = \frac{n}{n-1}\left(\frac{1}{n}\sum_{j=1}^{n}\pi_{ij}^2 - \left(\hat{p}_i^T\right)^2\right),$$

so substituting it into Eq. (S8) recovers Eq. (13) in the main text as desired:

$$\hat{F}_{\text{ST}}^{\text{indep}} = \frac{\sum_{i=1}^{m}\hat{\sigma}_i^2}{\sum_{i=1}^{m}\hat{p}_i^T\left(1-\hat{p}_i^T\right) + \frac{1}{n}\hat{\sigma}_i^2}.$$

C.2 Standard kinship estimator

Here we assume the kinship model in Eq. (5) in the main text. Since the first moment is the same for all individuals j, we average these genotypes to reduce variance,

$$\text{E}\left[\sum_{j=1}^{n} w_j x_{ij} \middle| T\right] = 2p_i^T,$$

which results in the following estimator of p_i^T:

$$\hat{p}_i^T = \frac{1}{2}\sum_{j=1}^{n} w_j x_{ij}.$$

Each φ_{jk}^T appears once per (j,k) pair in Eq. (5), recast here in terms of the sample covariance:

$$\text{E}\left[\left(x_{ij} - 2p_i^T\right)\left(x_{ik} - 2p_i^T\right) \middle| T\right] = 4p_i^T\left(1-p_i^T\right)\varphi_{jk}^T.$$

Variance in the kinship estimate is reduced by averaging across loci, yielding:

$$\text{E}\left[\frac{1}{m}\sum_{i=1}^{m}\left(x_{ij} - 2p_i^T\right)\left(x_{ik} - 2p_i^T\right) \middle| T\right] = 4\varphi_{jk}^T \frac{1}{m}\sum_{i=1}^{m} p_i^T\left(1-p_i^T\right). \tag{S9}$$

Plugging $\hat{p}_i^T$ into Eq. (S9) and solving for φ_{jk}^T recovers Eq. (18) in the main text as desired:

$$\hat{\varphi}_{jk}^{T,\text{std}} = \frac{\sum_{i=1}^{m}\left(x_{ij} - 2\hat{p}_i^T\right)\left(x_{ik} - 2\hat{p}_i^T\right)}{4\sum_{i=1}^{m}\hat{p}_i^T\left(1-\hat{p}_i^T\right)}.$$

D Proofs that $F_{\mathbf{ST}}$ and kinship estimator limits are constants with respect to the ancestral population T

In our work we calculate the limits of several estimators, which are given in terms of an arbitrary ancestral population T (not necessarily the MRCA, unless otherwise noted). The apparent paradox that the limit of an estimator would vary depending on the choice of T is resolved since these limits are in fact constant with respect to T. All proofs depend on the following IBD identities for change of ancestral population [10]:

$$\begin{aligned} \left(1-f_j^A\right) &= \left(1-f_j^B\right)\left(1-f_B^A\right), \\ \left(1-\varphi_{jk}^A\right) &= \left(1-\varphi_{jk}^B\right)\left(1-f_B^A\right), \end{aligned} \tag{S10}$$

where A, B are two possible ancestral populations for the individuals j, k, and A is ancestral to B.

D.1 Proof that the limit of $\hat{F}_{\mathbf{ST}}^{\mathbf{indep}}$ does not depend on T

Here we study the limit of $\hat{F}_{\text{ST}}^{\text{indep}}$ in Eq. (14) in the main text. Let S be a reference population ancestral to the individuals in question and T be another population ancestral to S. Denote the key parameters relative to S by $F_{\text{ST}}^S, \bar{\theta}^S$ and relative to T by $F_{\text{ST}}^T, \bar{\theta}^T$. The equations that relate both quantities satisfy our IBD shift identity (which follows by averaging Eq. (S10) over individuals for F_{ST} or pairs of individuals for $\bar{\theta}^T$):

$$\begin{aligned} \left(1-F_{\text{ST}}^T\right) &= \left(1-F_{\text{ST}}^S\right)\left(1-f_S^T\right), \\ \left(1-\bar{\theta}^T\right) &= \left(1-\bar{\theta}^S\right)\left(1-f_S^T\right). \end{aligned}$$

Solving for the values relative to S gives

$$F_{\text{ST}}^S = \frac{F_{\text{ST}}^T - f_S^T}{1-f_S^T}, \qquad \bar{\theta}^S = \frac{\bar{\theta}^T - f_S^T}{1-f_S^T}.$$

The desired equality of the limit for both S and T follows:

$$\begin{aligned} \frac{n\left(F_{\text{ST}}^S - \bar{\theta}^S\right)}{n-1+F_{\text{ST}}^S - n\bar{\theta}^S} &= \frac{n\left(\frac{F_{\text{ST}}^T - f_S^T}{1-f_S^T} - \frac{\bar{\theta}^T - f_S^T}{1-f_S^T}\right)}{n-1+\frac{F_{\text{ST}}^T - f_S^T}{1-f_S^T} - n\frac{\bar{\theta}^T - f_S^T}{1-f_S^T}} \\ &= \frac{n\left(F_{\text{ST}}^T - \bar{\theta}^T\right)}{(n-1)\left(1-f_S^T\right) + \left(F_{\text{ST}}^T - f_S^T\right) - n\left(\bar{\theta}^T - f_S^T\right)} \\ &= \frac{n\left(F_{\text{ST}}^T - \bar{\theta}^T\right)}{n-1+F_{\text{ST}}^T - n\bar{\theta}^T}. \end{aligned}$$

D.2 Proof that the limit of $\hat{\varphi}_{jk}^{T,\mathbf{std}}$ does not depend on T

Here we study the limit of the standard kinship estimator $\hat{\varphi}_{jk}^{T,\text{std}}$ in Eq. (19) in the main text. Let S be a reference population ancestral to the individuals in question and T be another population

ancestral to S. The equations that relate the terms relative to S and those relative to T follow from Eq. (S10) just as in the previous subsection:

$$\varphi_{jk}^S = \frac{\varphi_{jk}^T - f_S^T}{1 - f_S^T}, \qquad \bar{\varphi}_j^S = \frac{\bar{\varphi}_j^T - f_S^T}{1 - f_S^T},$$

$$\bar{\varphi}_k^S = \frac{\bar{\varphi}_k^T - f_S^T}{1 - f_S^T}, \qquad \bar{\varphi}^S = \frac{\bar{\varphi}^T - f_S^T}{1 - f_S^T}.$$

The desired result follows:

$$\frac{\varphi_{jk}^S - \bar{\varphi}_j^S - \bar{\varphi}_k^S + \bar{\varphi}^S}{1 - \bar{\varphi}^S} = \frac{\varphi_{jk}^T - \bar{\varphi}_j^T - \bar{\varphi}_k^T + \bar{\varphi}^T}{1 - \bar{\varphi}^T}.$$

E Mean coancestry bounds

Here we prove that, for any weights such that $w_j > 0$, $\sum_{j=1}^n w_j = 1$,

$$0 \le \bar{\theta}^T \le F_{\text{ST}} \le 1,$$

and for uniform weights $\frac{1}{n} F_{\text{ST}} \le \bar{\theta}^T$. Furthermore, $\bar{\theta}^T = F_{\text{ST}}$ iff $\theta_{jk}^T = F_{\text{ST}}$ for all (j, k), and $\bar{\theta}^T = \frac{1}{n} F_{\text{ST}}$ for the independent subpopulations model.

The Cauchy-Schwarz inequality for covariances implies $\theta_{jk}^T \le \sqrt{\theta_{jj}^T \theta_{kk}^T}$. Therefore,

$$\bar{\theta}^T = \sum_{j=1}^n \sum_{k=1}^n w_j w_k \theta_{jk}^T \le \left(\sum_{j=1}^n w_j \sqrt{\theta_{jj}^T} \right)^2 \le \sum_{j=1}^n w_j \theta_{jj}^T = F_{\text{ST}},$$

where the second inequality follows from Jensen's inequality, since x^2 is a convex function. Since $\theta_{jj}^T \le 1$, then $F_{\text{ST}} \le 1$ as well. Equality in the second bound requires $\theta_{jj}^T = F_{\text{ST}}$ for all j, and equality in the first bound requires $\theta_{jk}^T = \theta_{jj}^T = \theta_{kk}^T$, so that $\bar{\theta}^T = F_{\text{ST}}$ requires $\theta_{jk}^T = F_{\text{ST}}$ for all (j, k). Since all $w_j, \theta_{jk}^T \ge 0$, then

$$0 \le \sum_{j=1}^n w_j^2 \theta_{jj}^T \le \bar{\theta}^T,$$

where the second inequality follows from dropping $j \ne k$ terms from the double sum of $\bar{\theta}^T$. The case $w_j = \frac{1}{n}$ gives $\frac{1}{n} F_{\text{ST}} \le \bar{\theta}^T$, with equality for the independent subpopulations model by construction.

F Moments of estimator building blocks

Here we calculate first and some second moments for "building block" quantities that recur in our estimators, particularly terms involving x_{ij} and $\hat{p}_i^T$, and which enable us to calculate the limits of

our estimators. Below are examples for genotypes, which follow from Eq. (5) in the main text; calculations for IAFs follow analogously from Eq. (6) in the main text (not shown).

$$
\begin{aligned}
\mathrm{E}\left[\hat{p}_i^T | T\right] &= \mathrm{E}\left[\left.\frac{1}{2}\sum_{j=1}^n w_j x_{ij}\right| T\right] = \frac{1}{2}\sum_{j=1}^n w_j \,\mathrm{E}[x_{ij}|T] = \sum_{j=1}^n w_j p_i^T = p_i^T, \\
\mathrm{E}[x_{ij}x_{ik}|T] &= \mathrm{Cov}(x_{ij}, x_{ik}|T) + \mathrm{E}[x_{ij}|T]\,\mathrm{E}[x_{ik}|T] = 4\left(p_i^T\left(1-p_i^T\right)\varphi_{jk}^T + \left(p_i^T\right)^2\right), \\
\mathrm{E}\left[x_{ij}\hat{p}_i^T | T\right] &= \mathrm{E}\left[\left.\frac{1}{2}\sum_{k=1}^n w_j x_{ij} x_{ik}\right| T\right] = \frac{1}{2}\sum_{k=1}^n w_j \,\mathrm{E}[x_{ij}x_{ik}|T] \\
&= 2\sum_{k=1}^n w_j \left(p_i^T\left(1-p_i^T\right)\varphi_{jk}^T + \left(p_i^T\right)^2\right) = 2\left(p_i^T\left(1-p_i^T\right)\bar{\varphi}_j^T + \left(p_i^T\right)^2\right), \\
\mathrm{Var}\left(\hat{p}_i^T | T\right) &= \mathrm{Var}\left(\left.\frac{1}{2}\sum_{j=1}^n w_j x_{ij}\right| T\right) = \frac{1}{4}\sum_{j=1}^n\sum_{k=1}^n w_j w_k \,\mathrm{Cov}(x_{ij}, x_{ik}|T) = p_i^T\left(1-p_i^T\right)\bar{\varphi}^T, \\
\mathrm{E}\left[\left.\left(\hat{p}_i^T\right)^2\right| T\right] &= \mathrm{Var}\left(\hat{p}_i^T | T\right) + \mathrm{E}\left[\hat{p}_i^T | T\right]^2 = p_i^T\left(1-p_i^T\right)\bar{\varphi}^T + \left(p_i^T\right)^2, \\
\mathrm{E}\left[\hat{p}_i^T\left(1-\hat{p}_i^T\right) | T\right] &= \mathrm{E}\left[\hat{p}_i^T | T\right] - \mathrm{E}\left[\left.\left(\hat{p}_i^T\right)^2\right| T\right] = p_i^T\left(1-p_i^T\right)\left(1-\bar{\varphi}^T\right).
\end{aligned}
$$

G Derivation of new kinship estimator

To begin the method-of-moments derivation, we compute the raw first and second moments from the kinship model of Eq. (5) in the main text.

$$
\begin{aligned}
\mathrm{E}[x_{ij}|T] &= 2p_i^T, \\
\mathrm{E}[x_{ij}x_{ik}|T] &= \mathrm{E}[x_{ij}|T]\,\mathrm{E}[x_{ik}|T] + \mathrm{Cov}(x_{ij}x_{ik}|T) \\
&= 4\left(p_i^T\right)^2 + 4p_i^T\left(1-p_i^T\right)\varphi_{jk}^T.
\end{aligned}
$$

For obtain a symmetric estimator, we also compute the raw moments of $2 - x_{ij}$ (which counts the alternative allele):

$$
\begin{aligned}
\mathrm{E}[2 - x_{ij}|T] &= 2\left(1-p_i^T\right), \\
\mathrm{E}\left[(2-x_{ij})(2-x_{ik})|T\right] &= 4\left(1-p_i^T\right)^2 + 4p_i^T\left(1-p_i^T\right)\varphi_{jk}^T.
\end{aligned}
$$

If we solved for p_i^T using the first moment equations, we would recover the standard kinship estimator of Eqs. (17) and (18), so we shall avoid this strategy.

To proceed, we average the two second moment equations above. Note that

$$
\begin{aligned}
\frac{1}{2}\left(x_{ij}x_{ik} + (2-x_{ij})(2-x_{ik})\right) &= (1-x_{ij})(1-x_{ik}) + 1, \\
\frac{1}{2}\left(\left(p_i^T\right)^2 + \left(1-p_i^T\right)^2\right) &= \frac{1}{2} - p_i^T\left(1-p_i^T\right).
\end{aligned}
$$

Therefore, the symmetric estimator (which gives the same calculation if the reference allele is switched) is

$$\begin{aligned}
\mathrm{E}\left[(1-x_{ij})(1-x_{ik})+1|T\right] &= 2+4p_i^T\left(1-p_i^T\right)\left(\varphi_{jk}^T-1\right) \quad \Rightarrow \\
\mathrm{E}\left[(1-x_{ij})(1-x_{ik})-1|T\right] &= 4p_i^T\left(1-p_i^T\right)\left(\varphi_{jk}^T-1\right).
\end{aligned}$$

A genome-wide estimate is obtained by averaging the previous statistics across loci, resulting in

$$\begin{aligned}
A_{jk} &= \frac{1}{m}\sum_{i=1}^{m}(x_{ij}-1)(x_{ik}-1)-1, \\
\mathrm{E}\left[A_{jk}|T\right] &= \left(\varphi_{jk}^T-1\right)v_m^T, \quad \text{where} \\
v_m^T &= \frac{4}{m}\sum_{i=1}^{m}p_i^T\left(1-p_i^T\right).
\end{aligned}$$

The new kinship estimator follows from obtaining a consistent estimator of the limit of v_m^T as m goes to infinity, and applying it to solve for φ_{jk}^T in the above equation for the expectation of A_{jk}, as detailed in the main text.

H Mathematical equivalence between the proposed kinship estimator as described here and our original 2016 proposal

The new kinship estimator presented in the main text is algebraically equivalent to the version presented in our 2016 manuscript [9]. As notation differs, here we establish in detail the correspondence.

We begin by restating the preprint estimator in notation as close as possible in the original preprint, but with two variables renamed as they clash with our present notation. In the preprint, there are two steps to the final kinship estimator, analogous to the two steps in our updated presentation. The first step consisted of obtaining preadjusted kinship estimates that had a uniform bias, and in the second step the estimates are unbiased using the minimum preadjusted kinship estimate. The preadjusted estimator (referred to as $\hat{\varphi}_{jk}^{T,\text{new}}$ in the preprint, but which we will denote by C_{jk} here since it clashes with the notation of our final kinship estimator in Eq. (34) in the main text) was given by

$$C_{jk} = \frac{\sum_{i=1}^{m}(x_{ij}-1)(x_{ik}-1)-1}{4\sum_{i=1}^{m}\hat{p}_i^T(1-\hat{p}_i^T)}+1 \xrightarrow[m\to\infty]{\text{a.s.}} \frac{\varphi_{jk}^T-\bar{\varphi}^T}{1-\bar{\varphi}^T}. \tag{S11}$$

Thus, bias was determined by the unknown $\bar{\varphi}^T$, which plays the same role as the unknown v_m^T in our present work. The final estimator (denoted in the preprint by $\tilde{\varphi}_{jk}^{T,\text{new}}$, but which matches our

final kinship estimate $\hat{\varphi}_{jk}^{T,\text{new}}$ in Eq. (34) in the main text, so we use our new notation here) was given by

$$\hat{\varphi}_{jk}^{T,\text{new}} = C_{jk}(1 - \bar{\varphi}^T) + \bar{\varphi}^T \xrightarrow[m\to\infty]{\text{a.s.}} \varphi_{jk}^T. \tag{S12}$$

Lastly, it was proposed to estimate the unknown $\bar{\varphi}^T$ from the minimum C_{jk}, since under the hypothesis that the true minimum kinship is zero, then

$$\min_{jk} C_{jk} = \hat{C}_{\min} \approx C_{\min} = -\frac{\bar{\varphi}^T}{1 - \bar{\varphi}^T}. \tag{S13}$$

Lastly, the preprint recommends a more stable estimate than $\hat{C}_{\min}$ above. Thus, the general approach was in place that resulted in new kinship estimates given an estimator $\hat{C}_{\min}$ of $C_{\min}$.

Now we tie the preprint estimator to our present estimator. First note that Eq. (S11) is the same as

$$C_{jk} = \frac{A_{jk}}{B} + 1,$$

where A_{jk} is exactly as in Eq. (33) in the main text, and $B = 4\sum_{i=1}^m \hat{p}_i^T(1 - \hat{p}_i^T)$ is a constant shared by all individual pairs j and k. We will show that this B cancels out in the end, so that its form does not matter. Thus, in the present notation we eliminated B, resulting not only in a simpler presentation but also eliminating the only term in the preprint notation that depended on $\hat{p}_i^T$ (in the new formulation there is no need to estimate any allele frequencies).

The key is to notice that, since B is the same for all individual pairs, any approach to estimate the limit of the minimum A_{jk} corresponds directly to an approach for estimating the limit of the minimum C_{jk}, and vice versa, using the relation:

$$\hat{C}_{\min} = \frac{\hat{A}_{\min}}{B} + 1.$$

Solving for the mean kinship in Eq. (S13) results in an estimate given by

$$\hat{\bar{\varphi}}^T = -\frac{\hat{C}_{\min}}{1 - \hat{C}_{\min}} = \frac{\hat{A}_{\min} + B}{\hat{A}_{\min}}.$$

Lastly, plugging this $\hat{\bar{\varphi}}^T$ into Eq. (S12) results in a final estimator of

$$\begin{aligned}
\hat{\varphi}_{jk}^{T,\text{new}} &= C_{jk}(1 - \hat{\bar{\varphi}}^T) + \hat{\bar{\varphi}}^T \\
&= \left(\frac{A_{jk}}{B} + 1\right)\left(1 - \frac{\hat{A}_{\min} + B}{\hat{A}_{\min}}\right) + \frac{\hat{A}_{\min} + B}{\hat{A}_{\min}} \\
&= 1 - \frac{A_{jk}}{\hat{A}_{\min}},
\end{aligned}$$

which equals the present new estimator in Eq. (34) in the main text, as desired.

References

[1] H. B. Mann and A. Wald. "On Stochastic Limit and Order Relationships". *The Annals of Mathematical Statistics* 14(3) (1943), pp. 217–226.

[2] Patrick Billingsley. *Convergence of Probability Measures.* John Wiley & Sons, 2013. 247 pp.

[3] William Feller. *An introduction to probability theory and its applications.* 3rd ed. Vol. 1. John Wiley & Sons London-New York-Sydney-Toronto, 1968. 528 pp.

[4] H. O. Hartley and A. Ross. "Unbiased Ratio Estimators". *Nature* 174(4423) (1954), pp. 270–271.

[5] William Gemmell Cochran. *Sampling techniques.* 3rd ed. Wiley, 1977.

[6] Bruce S. Weir and Jérôme Goudet. "A Unified Characterization of Population Structure and Relatedness". *Genetics* 206(4) (2017), pp. 2085–2103.

[7] B. S. Weir and W. G. Hill. "Estimating F-Statistics". *Annual Review of Genetics* 36(1) (2002), pp. 721–750.

[8] John Buckleton et al. "Population-specific FST values for forensic STR markers: A worldwide survey". *Forensic Science International: Genetics* 23 (2016), pp. 91–100.

[9] Alejandro Ochoa and John D. Storey. "F_{ST} and kinship for arbitrary population structures II: Method of moments estimators". *bioRxiv* (10.1101/083923) (2016). `https://doi.org/10.1101/083923`.

[10] Alejandro Ochoa and John D. Storey. "F_{ST} and kinship for arbitrary population structures I: Generalized definitions". *bioRxiv* (10.1101/083915) (2016). `https://doi.org/10.1101/083915`.
